# Atractylenolide I stabilizes HMOX1 to induce ferroptosis and enhance lenvatinib efficacy in hepatocellular carcinoma

**DOI:** 10.1016/j.isci.2026.117259

**Published:** 2026-08-21

**Authors:** Yangyang Miao, Shengjie Jin, Rui Peng, Daoyuan Tu, Jiahao Zhang, Songsong Fan, Bingbing Su, Anyang Jiang, Jihu Zheng, Guoqing Jiang, Chi Zhang, Jun Cao, Dousheng Bai

**Affiliations:** 1 Department of General Surgery, Northern Jiangsu People’s Hospital Affiliated to Yangzhou University, Yangzhou 225001, China; 2 General Surgery Institute of Northern Jiangsu People’s Hospital, Yangzhou 225001, China; 3 The Yangzhou Clinical Medical College of Xuzhou Medical University, Xuzhou 221004, China; 4 The Changshu Hospital Affiliated to Nantong University, Suzhou 215500, China

**Keywords:** atractylenolide I, ferroptosis, heme oxygenase-1, HMOX1, hepatocellular carcinoma, lenvatinib, ubiquitination

## Abstract

Lenvatinib resistance limits the therapeutic efficacy of hepatocellular carcinoma (HCC), highlighting the need for effective strategies to enhance treatment response. Atractylenolide I (AT-1), a bioactive compound derived from *Atractylodes macrocephala*, exhibits antitumor activity, yet its mechanism in HCC remains unclear. Here, we demonstrate that AT-1 suppresses HCC growth by inducing ferroptosis and enhances Lenvatinib efficacy. AT-1 increases intracellular Fe^2+^ accumulation, lipid peroxidation, and reactive oxygen species, consistent with ferroptotic cell death. Mechanistically, AT-1 directly binds to heme oxygenase-1 (HMOX1) and stabilizes its protein expression by inhibiting ubiquitination at lysine residues K177 and K179, thereby preventing proteasomal degradation. Genetic or pharmacological inhibition of HMOX1 abrogates AT-1-induced ferroptosis and antitumor effects *in vitro* and *in vivo*. Notably, AT-1 synergistically enhances Lenvatinib-mediated tumor suppression in HCC models. These findings identify HMOX1 stabilization as a core ferroptosis-regulating mechanism and support AT-1 as a promising adjuvant strategy for HCC therapy.

## Highlights

• Atractylenolide I suppresses growth and migration of liver cancer cells
• Atractylenolide I stabilizes heme oxygenase one protein in liver cancer
• Atractylenolide I reinforces the anti-tumor effect of Lenvatinib in liver cancer

## Graphical abstract

**Figure:**
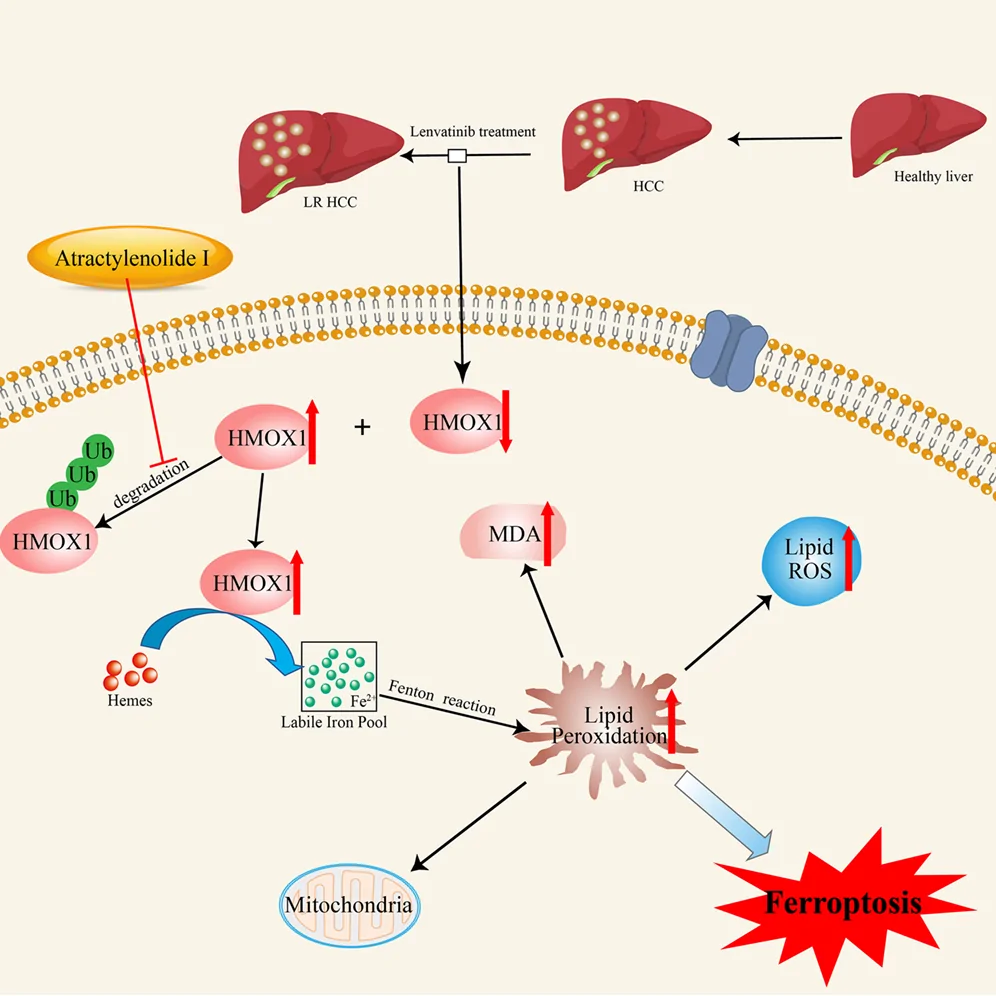

## Teaser

Biological sciences; Molecular biology

## Introduction

Hepatocellular carcinoma (HCC) is a leading cause of cancer-related mortality worldwide, accounting for approximately 85%–95% of primary liver cancers and exhibiting a poor 5-year overall survival of ∼18% despite advances in care.^1^^,^^2^^,^^3^ Although surgical resection and liver transplantation offer curative options for early-stage disease, most patients present at advanced stages when surgery is not feasible.^4^ Systemic agents such as sorafenib and Lenvatinib have improved outcomes in advanced HCC, yet efficacy is often transient, and intrinsic/acquired chemoresistance limits durable benefit.^5^ Thus, identifying viable therapeutic targets and efficacious pharmacological agents remains a clinical priority.

Recent advancements in imaging technologies, including photoacoustic and biomedical optics, have facilitated more precise, non-invasive monitoring of treatment efficacy and tumor dynamics.^6^^,^^7^ These techniques have become increasingly integrated into cancer therapy, providing valuable insights into tumor response and the potential for combination therapies.^8^ In this context, the combination of tractable ferroptosis inducers like atractylenolide I (AT-1) with conventional agents such as Lenvatinib could offer promising therapeutic strategies for HCC treatment, potentiating anti-tumor efficacy while supporting feasible therapeutic monitoring.

Modern phytochemistry has uncovered diverse bioactive compounds from traditional herbal resources, some with multi-target, multi-pathway activities reminiscent of classical formulas.^9^ *Atractylodes macrocephala* (Baizhu), a medicinal rhizome of the Asteraceae family, has long been used for its spleen-strengthening and qi-tonifying properties and displays immunomodulatory, anti-inflammatory, anti-cancer, and neuroprotective activities in pharmacologic studies.^10^^,^^11^^,^^12^ Its principal constituents include volatile oils, polysaccharides, and sesquiterpene lactones (atractylenolides I/II/III). Among these, AT-1 has garnered attention for anti-tumor activity^13^: in breast cancer, AT-1 suppressed proliferation and migration and induced apoptosis via TLR4/NF-κB inhibition^14^; in prostate cancer, it downregulated HSP27 and enhanced the efficacy of cabozantinib^15^; and it inhibited cervical cancer cell growth, with further effects reported in combination settings. Despite these advances, the targets and regulatory networks of AT-1 in HCC remain unclear.

Ferroptosis is an iron-dependent regulated cell death characterized by lipid peroxidation resulting from iron overload and excessive lipid hydroperoxides.^16^^,^^17^ It has been implicated in neurodegeneration, ischemia-reperfusion injury, and cancer,^18^^,^^19^^,^^20^^,^^21^^,^^22^ and is increasingly recognized as a therapeutic axis in HCC,^23^ intersecting with multiple oncogenic pathways. Certain malignancies exhibit heightened ferroptosis susceptibility due to metabolic reprogramming, ROS propensity, and driver mutations, opening avenues for precision therapy.^24^^,^^25^^,^^26^ Heme oxygenase-1 (HMOX1), a stress-responsive enzyme that degrades heme into biliverdin, carbon monoxide, and ferrous iron (Fe^2+^), occupies a central position at the interface of redox and iron metabolism.^27^ While physiological HMOX1 supports homeostasis, pathological upregulation can perturb iron handling and promote cell death in a dose-dependent manner.^28^^,^^29^^,^^30^ These observations motivate an investigation into how HMOX1-ferroptosis crosstalk might be therapeutically leveraged in HCC.

Here, we show that AT-1 exerts anti-HCC effects by inducing ferroptosis through HMOX1 stabilization. Functionally and mechanistically, AT-1 prolonged HMOX1 protein half-life and reduced its ubiquitination, with K177/K179 lysines functionally implicated in this process, thereby expanding labile Fe^2+^ pools and triggering GPX4-independent ferroptosis. *In vitro* and *in vivo*, AT-1 inhibited tumor growth and, importantly, enhanced the antitumor activity of Lenvatinib. These findings nominate HMOX1 as a tractable node for HCC therapy and position AT-1 as a ferroptosis-enabling agent and rational partner for Lenvatinib.

## Results

### AT-1 suppresses HCC cell growth and migration

**Figure 1:**
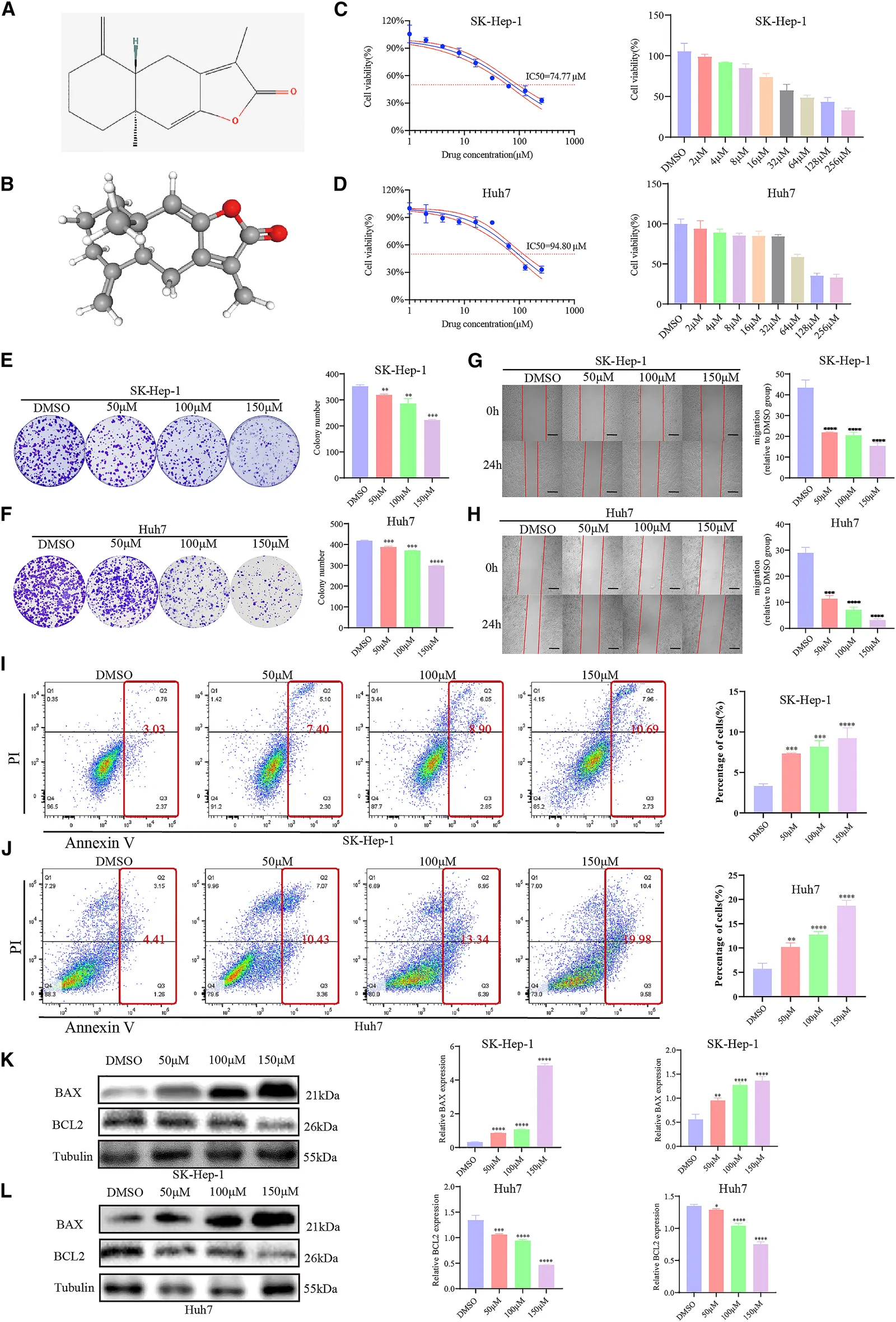
AT-1 suppresses HCC cell growth, migration and promotes apoptosis (A) 2D chemical structure of AT-1. (B) 3D structural model of AT-1. (C and D) Dose-response curves and IC_50_ determination for SK-Hep-1 and Huh7 cells. Cells were seeded in clear-bottom 96-well plates (2,000 cells/well), treated with AT-1 (0–256 μM, 24 h), incubated with 10% (v/v) CCK-8 for 1 h, and viability was normalized to vehicle (DMSO). IC_50_ values were obtained by four-parameter logistic (4 PL) nonlinear regression. (E and F) Clonogenic growth. Cells (2 × 10^3^/well) were plated in 6-well plates, exposed to the indicated concentrations of AT-1 in medium containing 20% FBS for 14 days, fixed/stained, colonies counted, and quantified. (G and H) Wound-healing migration. Confluent monolayers were scratched and treated with AT-1; wound areas were imaged at 0 h and 24 h and quantified as % closure relative to 0 h. (I and J) Apoptosis analysis. SK‑Hep‑1 and Huh7 cells were treated with AT‑1; apoptosis ratios were assessed by flow cytometry, and results were statistically analyzed. (K and L) Western blots of BAX and BCL2 in SK-Hep-1 and Huh7 cells treated with AT-1; Tubulin serves as loading control, with densitometric quantification shown. Data are mean ± SEM from independent experiments (*n* ≥ 3). Statistical: one-way ANOVA with Dunnett’s post-hoc test vs. DMSO for multi-dose comparisons; two-way ANOVA for time-course data (G–H); two-tailed unpaired *t* tests where appropriate. Significance: ∗*p* < 0.05, ∗∗*p* < 0.01, ∗∗∗*p* < 0.001, ∗∗∗∗*p* < 0.0001. Scale bars, 200 μm.

Atractylenolides are sesquiterpene lactones from *A. macrocephala* that comprise three structural isomers (AT-1/-2/-3); among them, AT-1 exhibits the strongest anti-tumor activity (Figures 1A and 1B). To assess its effects on proliferation, SK-Hep-1 and Huh7 cells were exposed to increasing AT-1 concentrations (0, 2, 4, 8, 16, 32, 64, 128, and 256 μM) for 24 h. Cell viability (CCK-8) decreased in a concentration-dependent manner, yielding IC_50_ values of 74.8 μM (SK-Hep-1) and 94.8 μM (Huh7) (Figures 1C and 1D). Guided by these values, 50, 100, and 150 μM were selected for subsequent assays. Consistently, colony formation was markedly reduced in both lines (Figures 1E and 1F). In wound-healing assays performed in serum-reduced medium to minimize proliferation confounding, AT-1 significantly slowed closure of scratched monolayers (Figures 1G and 1H).

AT-1 also increased apoptosis in a dose-dependent fashion, as determined by annexin V-FITC/PI flow cytometry (Figures 1I and 1J). Western blotting corroborated these findings, showing BAX upregulation with BCL-2 downregulation after AT-1 treatment (Figures 1K and 1L).

To evaluate selectivity, non-transformed human hepatocytes (THLE-2) were examined. AT-1 displayed low cytotoxicity toward THLE-2 (IC_50_ ≈ 172.8 μM); viability was largely preserved at ≤100 μM and declined appreciably only at ≥128 μM (Figures S1A and S1B). These data indicate a therapeutic window in which anti-HCC activity is achieved with minimal impact on normal hepatocytes.

### AT-1 suppresses HCC cell proliferation *in vivo*

**Figure 2:**
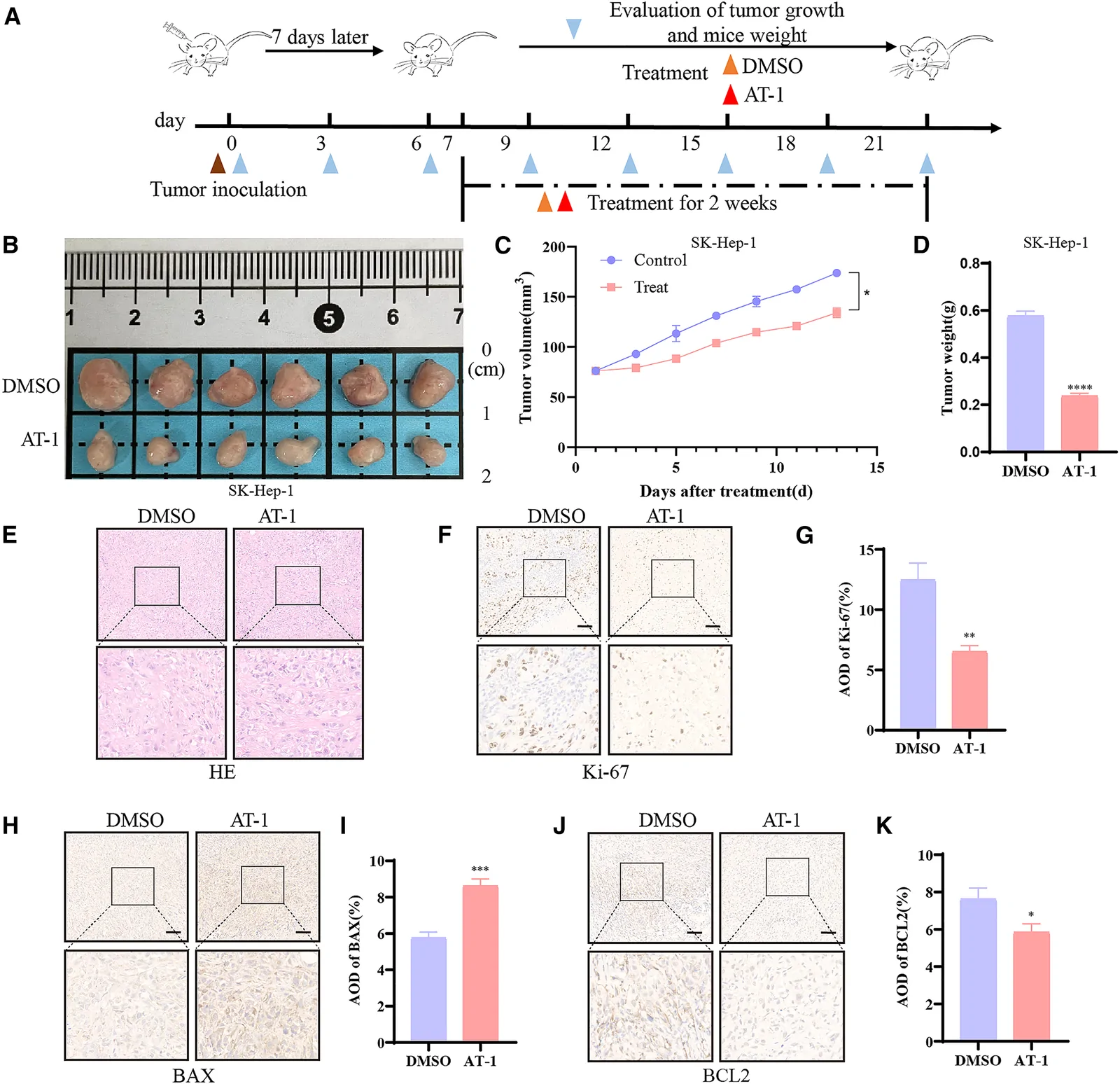
AT-1 suppresses HCC cell proliferation *in vivo* (A) Experimental timeline for subcutaneous xenografts established with wild-type SK-Hep-1 cells in nude mice. Beginning 7 days after inoculation, mice received AT-1 (25 mg/kg, i.p., once daily) for 14 consecutive days; tumor size and body weight were monitored every 3 days. (B–E) Representative photographs of excised tumors (B), tumor-growth curves (C; mean ± SEM), and endpoint tumor weights (D; mean ± SEM) for vehicle (DMSO) vs. AT-1 groups (*n* = 6 per group). Tumor volume was calculated as V = (L × W^2^)/2 (E), hematoxylin and eosin (H&E) staining of tumor sections. (F–K) Immunohistochemistry (IHC) for Ki-67, BAX, and BCL2. Left, representative images; right, quantitative analysis shown as average optical density (AOD). Statistics: two-way ANOVA for growth curves (C) and two-tailed unpaired *t* tests for tumor weight and IHC quantification (D, G, I, and K). Significance: ∗*p* < 0.05, ∗∗*p* < 0.01, ∗∗∗*p* < 0.001, ∗∗∗∗*p* < 0.0001. Scale bars, 100 μm.

To determine whether the *in-vitro* activity of AT-1 translates *in vivo*—and whether efficacy persists in an immune-competent setting—we evaluated AT-1 in two murine models: a human SK-Hep-1 xenograft and a Hep1-6 syngeneic allograft in C57BL/6 mice (Figure 2A). Once tumors were palpable, mice (*n* = 6 per group) were randomized to vehicle (DMSO) or AT-1. The AT-1 cohort received intraperitoneal injections of 25 mg/kg once daily for 14 consecutive days (i.p., qd × 14 days); vehicle recipients received matched-volume DMSO. At study end, AT-1 significantly reduced tumor volume, slowed growth kinetics, and decreased tumor weight relative to vehicle (Figures 2B–2D and S1C–S1E). Body weight remained stable, and no abnormal behaviors were observed throughout the experiment. Consistent with the pro-apoptotic profile of AT-1, tumor immunohistochemistry (IHC) showed BCL-2 and Ki-67 downregulation with BAX upregulation (Figures 2E–2G).

To assess systemic safety, hematoxylin and eosin (H&E) staining and IHC of the heart, liver, spleen, lung, and kidney were performed at study termination in the SK-Hep-1 xenograft models. No overt histopathological lesions (e.g., necrosis, hemorrhage, inflammatory-cell infiltration, or fibrosis) or abnormal IHC patterns were detected in AT-1-treated animals versus controls (Figures S1F–S1J). Together with stable body weight and normal behavior, these findings support the *in-vivo* tolerability of AT-1. Collectively, the *in-vivo* data corroborate the anti-proliferative and pro-apoptotic effects of AT-1 in HCC.

### AT-1 induces ferroptosis in HCC cells

**Figure 3:**
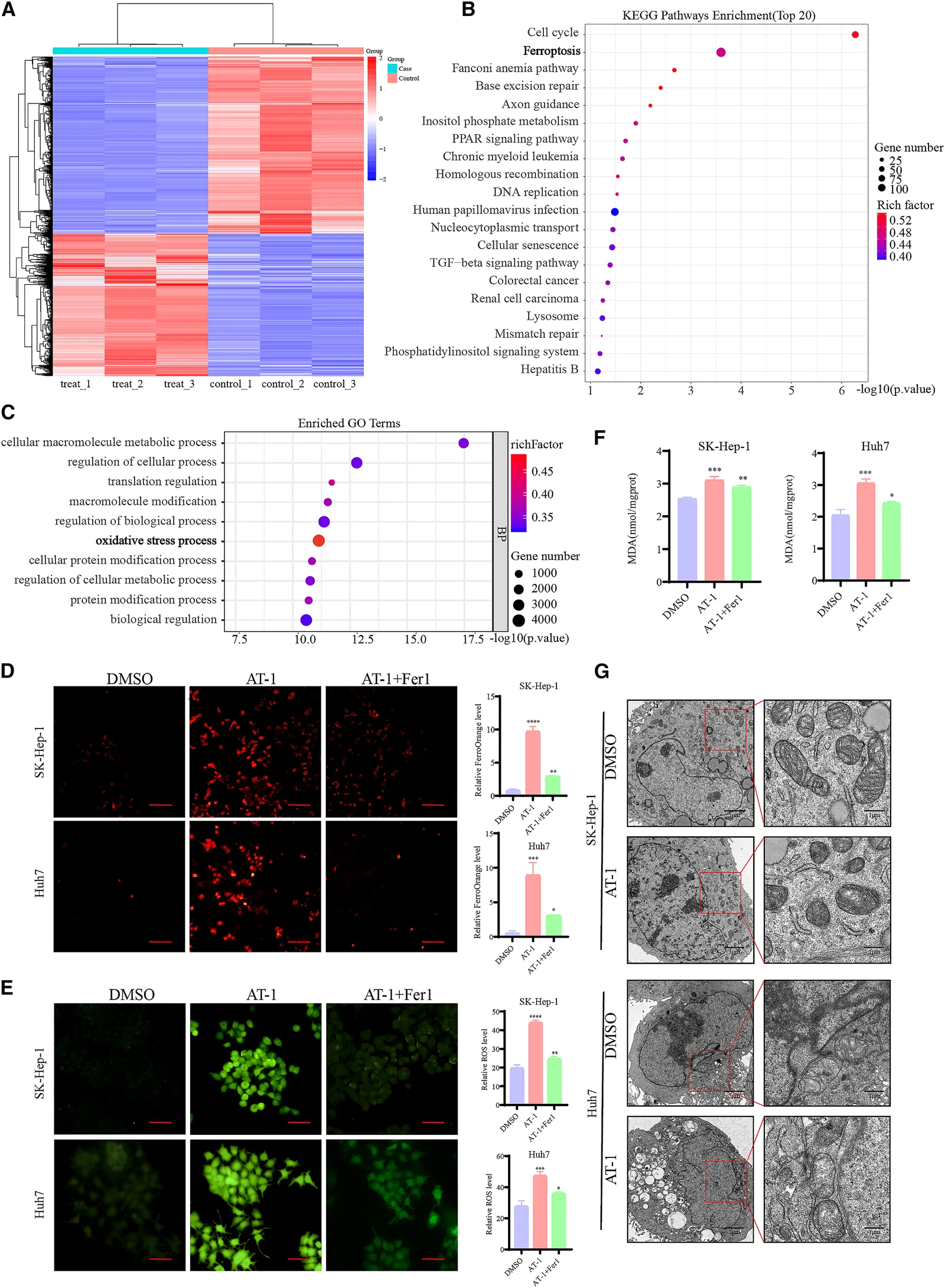
AT-1 induces ferroptosis in HCC cells (A) RNA-seq heatmap showing 5,746 differentially expressed genes (DEGs) between DMSO and AT-1-treated cells (*n* = 3 per group; adjusted *p* < 0.05). (B and C) KEGG and Gene Ontology (GO) enrichment of DEGs, highlighting ferroptosis and oxidative-stress-related terms. (D) FerroOrange staining of labile Fe^2+^ in SK-Hep-1 and Huh7 cells after 24 h treatment with DMSO, AT-1, or AT-1 + ferrostatin-1 (Fer-1); representative fluorescence images and mean fluorescence intensity (MFI) quantification are shown. (E) DCFH-DA detection of intracellular ROS under the same conditions as in (D), with representative images and MFI quantification. (F) Malondialdehyde (MDA) levels measured after 24 h treatments as in (D), normalized and quantified. (G) Transmission electron microscopy (TEM) images after 24 h DMSO or AT-1, illustrating ferroptotic mitochondrial features (condensed mitochondria with reduced/vanished cristae and increased membrane density); right panels show magnified insets of boxed regions. Data are mean ± SEM from ≥3 independent experiments. Statistics: one-way ANOVA with Tukey’s multiple-comparisons test for multi-group analyses (D–F). Significance: ∗*p* < 0.05, ∗∗*p* < 0.01, ∗∗∗*p* < 0.001, ∗∗∗∗*p* < 0.0001. Scale bars, 100 μm in (D) and (E); 5 μm in (G).

Guided by the IC_50_ values for SK-Hep-1 (74.8 μM) and Huh7 (94.8 μM), 100 μM AT-1 was used for mechanistic studies. Transcriptomic profiling revealed 5,746 differentially expressed genes (DEGs)—2,558 upregulated and 3,188 downregulated in AT-1-treated versus control cells (Figure 3A). KEGG and Gene Ontology (GO) enrichment analyses converged on ferroptosis and oxidative stress-related pathways (Figures 3B and 3C), suggesting disruption of redox homeostasis and activation of iron-dependent cell death programs.

Consistent with ferroptosis biomarker criteria,^31^ FerroOrange staining demonstrated an accumulation of labile Fe^2+^ after AT-1 exposure, which was partially reversed by ferrostatin-1 (Fer-1) (Figure 3D). AT-1 also increased ROS levels measured by DCFH-DA fluorescence in both SK-Hep-1 and Huh7 cells, and this effect was significantly blunted by Fer-1 (Figure 3E). In parallel, malondialdehyde (MDA)—a lipid peroxidation readout—was elevated by AT-1 and reduced by Fer-1 co-treatment (Figure 3F). Transmission electron microscopy further revealed canonical ferroptotic ultrastructures in AT-1-treated cells, including mitochondrial shrinkage, condensed membranes, loss/fragmentation of cristae, and occasional outer-membrane rupture (Figure 3G).

Collectively, these data demonstrate that AT-1 suppresses HCC cell growth by inducing ferroptosis, as evidenced by Fe^2+^ overload, ROS and lipid peroxidation, and characteristic mitochondrial remodeling, with Fer-1 rescue supporting pathway specificity.

### AT-1 mediates ferroptosis in HCC cells via upregulation of HMOX1 expression

**Figure 4:**
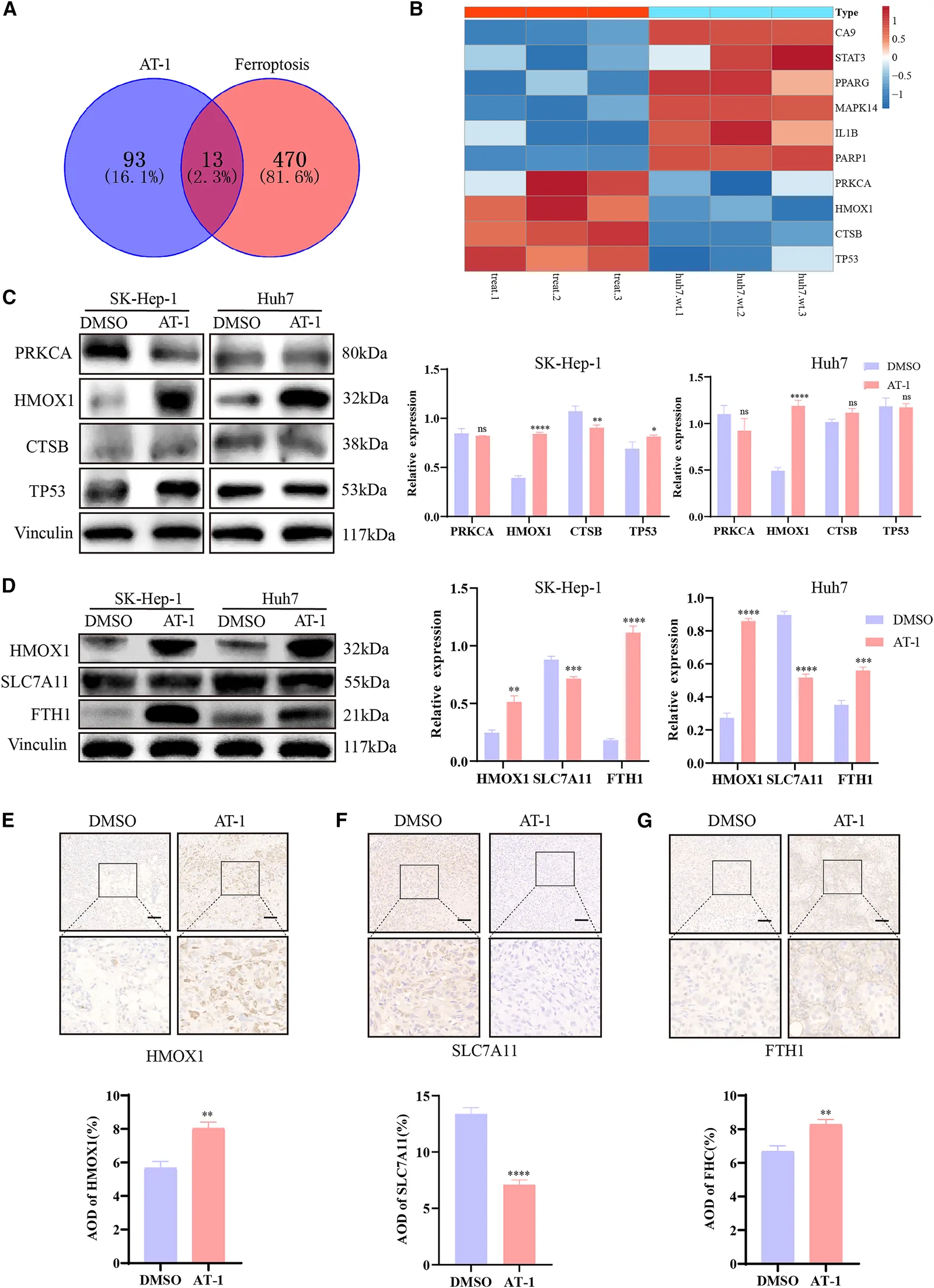
AT-1 upregulates HMOX1 and reshapes ferroptosis markers in HCC cells (A) Venn diagram showing the overlap between predicted AT-1 targets and a curated ferroptosis gene set, yielding 13 candidates. (B) Heatmap of these 13 genes from RNA-seq comparing AT-1 vs. DMSO (*n* = 3 per group; *Z* score-scaled expression), highlighting changes in genes such as HMOX1, PRKCA, CTSB, and TP53. (C) Immunoblot analysis of proteins encoded by selected DEGs in SK-Hep-1 and Huh7 cells after DMSO or AT-1 treatment; band intensities were quantified and normalized to Vinculin. (D) Immunoblots of ferroptosis-related regulators showing HMOX1 increased, SLC7A11 decreased, and FTH1 (ferritin heavy chain 1) increased upon AT-1 treatment in both cell lines; densitometric quantification is shown. (E–G) Immunohistochemistry (IHC) of xenograft tumors for HMOX1, SLC7A11, and FTH1; representative images (top) and AOD (average optical density) quantification (bottom). IHC results are consistent with the *in-vitro* findings (HMOX1↑, SLC7A11↓, and FTH1↑). Data are presented as mean ± SEM from ≥3 independent experiments. Statistics: two-tailed unpaired *t* tests for pairwise comparisons (C–G). Significance: ∗*p* < 0.05, ∗∗*p* < 0.01, ∗∗∗*p* < 0.001, ∗∗∗∗*p* < 0.0001. Scale bars, 100 μm.

To identify putative targets of AT-1, we intersected AT-1-associated genes predicted by SwissTargetPrediction, HERB, and PubChem (*n* = 93) with ferroptosis-related genes curated in FerrDb (*n* = 470). The overlap comprised 13 candidates (Figure 4A). RNA-seq comparing AT-1-treated versus DMSO controls revealed 10 DEGs among these candidates; PRKCA, HMOX1, CTSB, and TP53 were upregulated in a pattern consistent with ferroptosis activation (Figure 4B). Western blotting confirmed that HMOX1 protein—but not PRKCA, CTSB, or TP53—was robustly increased by AT-1, concordant with its mRNA change (Figure 4C), nominating HMOX1 as a key mediator of AT-1 action.

To validate this hypothesis, we profiled ferroptosis-pathway components downstream of HMOX1. AT-1 upregulated HMOX1 and decreased SLC7A11 (the cystine/glutamate antiporter controlling cystine uptake and glutathione synthesis), while increasing FTH1 (iron-storage heavy chain) (Figure 4D). GPX4 levels remained essentially unchanged (Figure S2A), aligning with a GPX4-independent ferroptosis mechanism.

Mechanistically, HMOX1 degrades heme to biliverdin, carbon monoxide, and free iron, thereby enlarging labile Fe^2+^ pools. Excess Fe^2+^ fuels Fenton chemistry, generating ROS and lipid hydroperoxides that can overwhelm antioxidant defenses even when GPX4 is intact.^29^^,^^32^ This “Fe^2+^ overflow” constitutes a hallmark of non-canonical, GPX4-independent ferroptosis, wherein iron dyshomeostasis directly drives membrane lipid peroxidation and cell death.^33^

Corroborating the *in-vitro* data, IHC on xenograft tumors showed a higher fraction of HMOX1-positive cells in AT-1-treated mice versus controls (Figure 4E). In parallel, SLC7A11 staining decreased, whereas FTH1 increased (Figures 4F and 4G). By contrast, GPX4 expression was stable or only minimally changed (Figure S2B). The concordance between *in-vitro* WB and *in-vivo* IHC—particularly the absence of GPX4 downregulation—supports a model in which HMOX1 is a central mediator of AT-1-induced, GPX4-independent ferroptosis in HCC.

### AT-1 forms a stable interaction with the HMOX1 protein

**Figure 5:**
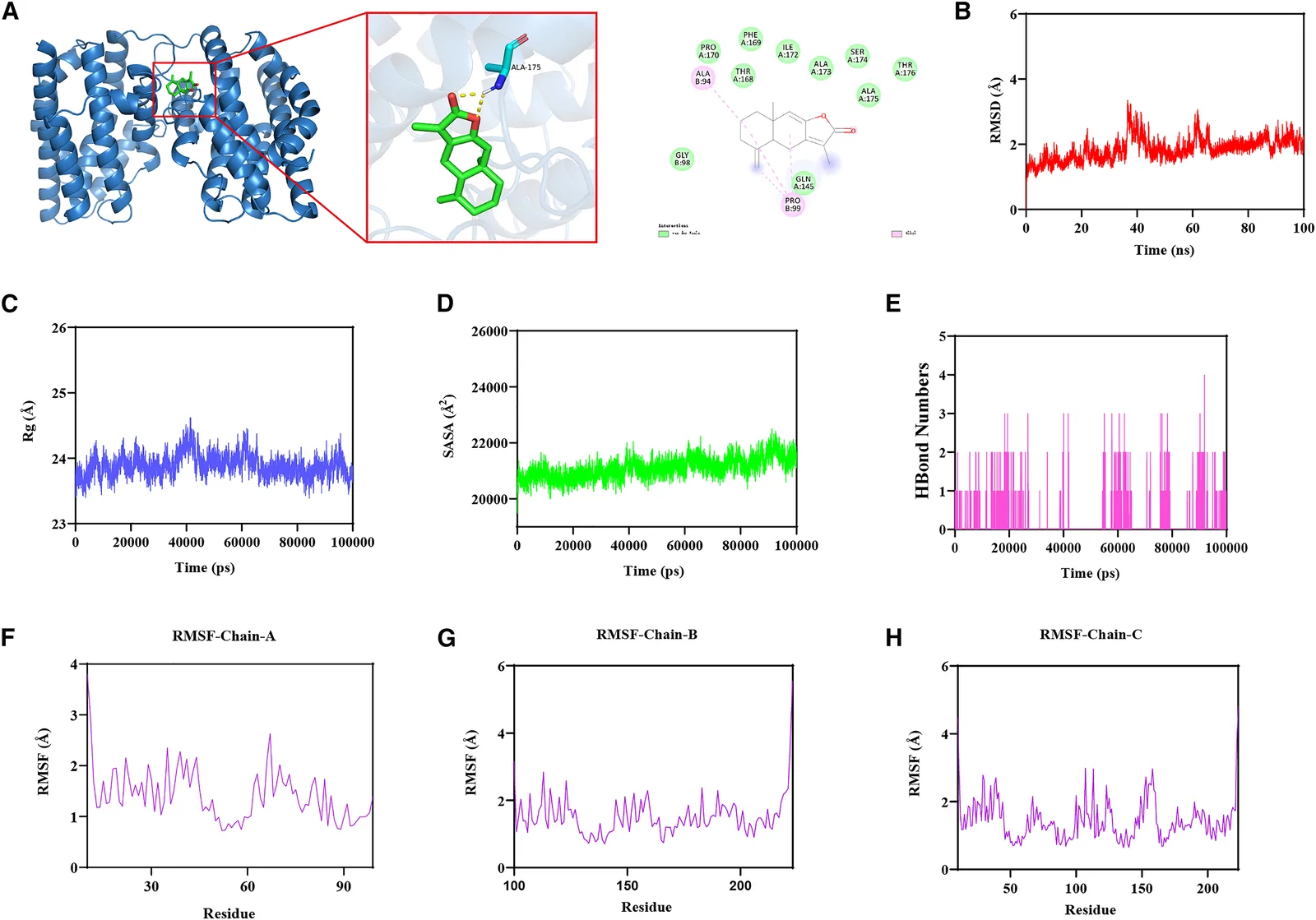
AT-1 forms a stable binding mode with HMOX1 supported by 100-ns MD (A) Overall binding conformation of AT-1 (green) with the HMOX1 protein (blue), showing detailed binding site interactions including hydrogen bonds (yellow dashed lines) and hydrophobic interactions between AT-1 and the active center of HMOX1. The chemical structure of the ligand AT-1 and its key functional groups are also displayed, with pink-labeled hydrophilic groups and green-labeled hydrophobic groups, demonstrating complementary adaptation to the protein’s active pocket. (B) Root-mean-square deviation (RMSD) curve of the complex over simulation time (0–100 ns) shows that the complex reached stability after 65 ns, with the final RMSD stabilizing near 2.2 Å, indicating dynamic equilibrium and high thermodynamic stability of the binding. (C) Radius of gyration (Rg) curve shows minimal fluctuations in the overall size of the complex during simulation (23–25 Å), suggesting a compact and stable conformation. (D) Solvent-accessible surface area (SASA) curve indicates low-level fluctuations in solvent-exposed area (approximately 22,000–24,000 Å^2^), confirming minimal solvent interference with the binding interface and maintenance of the functional spatial conformation. (E) Hydrogen bond number between AT-1 and HMOX1 oscillates primarily within the range of 0–4, with typical conformations maintaining approximately 2 hydrogen bonds, demonstrating stable binding via continuous and efficient hydrogen bonding interactions. (F–H) Root-mean-square fluctuation (RMSF) plots for three polypeptide chains (A, B, and C chains) show low RMSF values, indicating highly stable protein conformations and reduced non-specific fluctuations.

To evaluate whether AT-1 can stably bind HMOX1, we combined molecular docking with all-atom molecular dynamics (MD) simulations. Docking predicted a plausible pose in which AT-1 engages the HMOX1 pocket through hydrogen bonds and hydrophobic contacts (Figure 5A). In subsequent 100-ns MD simulations, the protein-ligand RMSD reached a plateau by ∼65 ns and stabilized near 2.2 Å, indicating a conformationally stable complex under the simulation conditions (Figure 5B).

Global shape and solvation metrics were consistent with a compact, well-behaved complex: both the radius of gyration (Rg) and solvent-accessible surface area (SASA) displayed only minor fluctuations over time (Figures 5C and 5D). Throughout the trajectory, the number of intermolecular hydrogen bonds fluctuated between 0 and 4 with a median of ∼2, reflecting a persistent H-bonding network that complements hydrophobic packing at the interface (Figure 5E).

Residue-level dynamics further supported a restrained binding environment. RMSF analysis showed generally low flexibility across the protein, with most residues exhibiting RMSF <3 Å (Figures 5F–5H), while loop regions displayed modest mobility typical of solvent-exposed segments.

Taken together, these *in-silico* analyses support a stable, persistent interaction between AT-1 and HMOX1, providing a structural rationale for the observed biochemical stabilization of HMOX1 upon AT-1 treatment.

### AT-1 enhances HMOX1 protein stability by inhibiting ubiquitination

**Figure 6:**
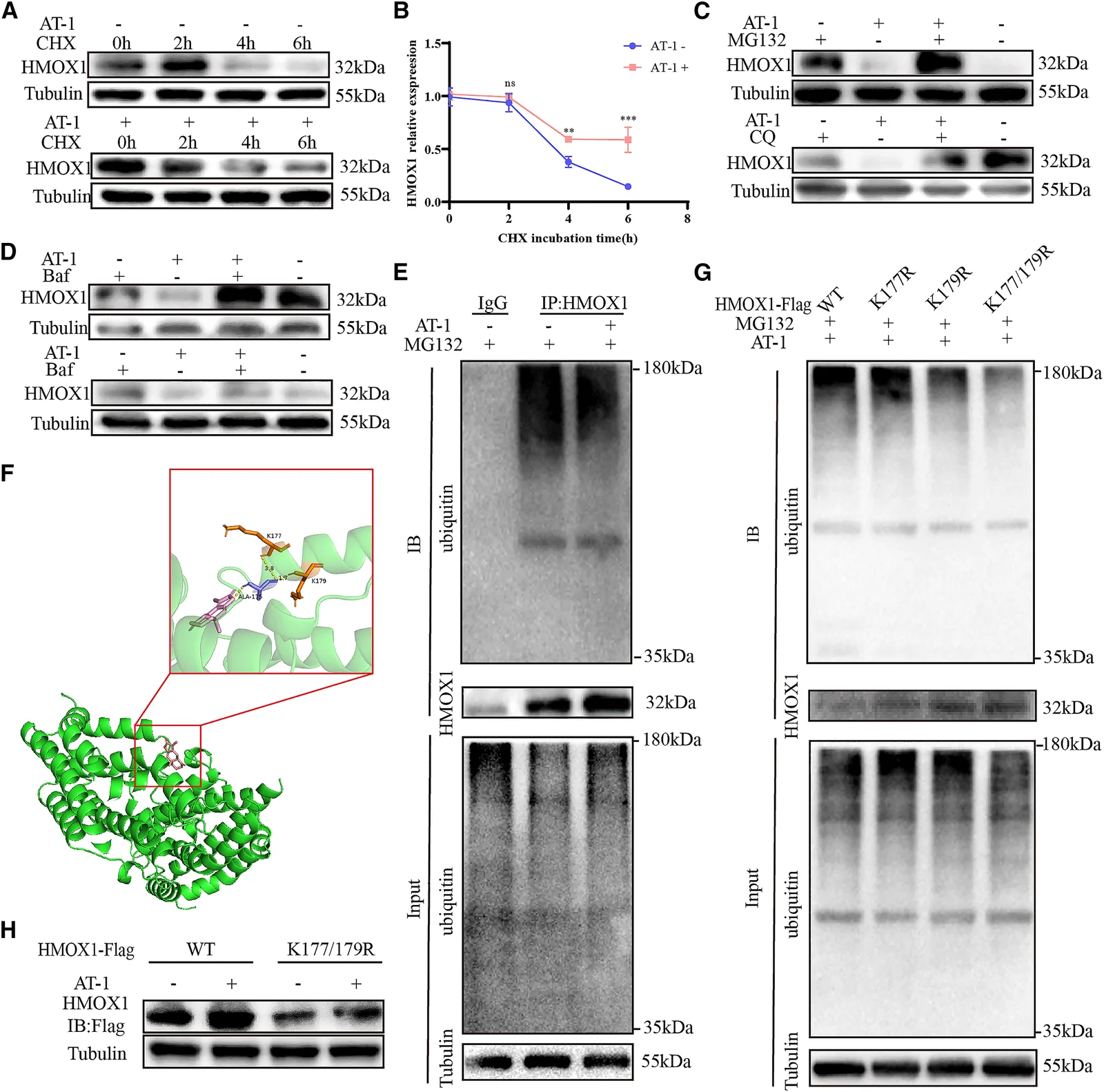
AT-1 stabilizes HMOX1 by blocking k177/k179 ubiquitination and proteasomal degradation (A) CHX chase in SK-Hep-1 cells with or without AT-1 showing HMOX1 decay over time; Tubulin serves as loading control. (B) Quantification of HMOX1 remaining (normalized to 0 h) indicates a prolonged half-life in the AT-1 group. (C and D) Pathway assignment using inhibitors: pretreatment with the proteasome inhibitor MG132 or lysosomal inhibitors chloroquine (CQ)/bafilomycin A1 (Baf) followed by AT-1. MG132 phenocopied/occluded the stabilizing effect of AT-1, whereas CQ/Baf had minimal impact, supporting proteasome-mediated turnover of HMOX1. (E) Ubiquitination assay. Following MG132 enrichment, HMOX1 was immunoprecipitated and probed for ubiquitin. AT-1 reduced polyubiquitinated HMOX1 smears, indicating decreased ubiquitination. (F) Structural model positioning Lys177/Lys179 adjacent to the AT-1 pocket, suggesting that ligand binding may hinder ubiquitin conjugation at these sites. (G) Mutational analysis of HMOX1 (K177R, K179R, K177/179R): ubiquitination of the mutants was diminished, and the ability of AT-1 to further reduce ubiquitination was attenuated. (H) Protein stability of HMOX1-Flag WT vs. K177/179R. The double mutant displayed higher basal abundance and was largely insensitive to AT-1-induced stabilization. Cells were transfected for 48 h and treated ± AT-1 for 12 h before analysis. Data are mean ± SEM (≥3 independent experiments). Statistics: two-way ANOVA for CHX chase (B); one-way ANOVA with Dunnett/Tukey for inhibitor and mutant comparisons (C, D, G, and H). Significance: ∗*p* < 0.05, ∗∗*p* < 0.01, ∗∗∗*p* < 0.001, ∗∗∗∗*p* < 0.0001.

Because ferroptosis depends on the levels of its effector proteins, we hypothesized that AT-1 increases the stability of HMOX1. In a cycloheximide (CHX) chase, AT-1 prolonged the half-life of HMOX1 relative to vehicle (Figures 6A and 6B), indicating reduced turnover. To define the degradation route, we treated cells with inhibitors of lysosomes (CQ or BafA1), autophagy (3-MA), or the proteasome (MG132) with or without AT-1. Only MG132 abolished the AT-1-induced increase in HMOX1 in both Huh7 and SK-Hep-1 cells (Figures 6C and 6D), implicating the ubiquitin-proteasome system (UPS) as the predominant pathway of HMOX1 turnover under basal conditions.

To directly test whether AT-1 affects HMOX1 ubiquitination, we immunoprecipitated HMOX1 and immunoblotted for ubiquitin. AT-1 reduced HMOX1 poly-ubiquitination in SK-Hep-1 cells (Figure 6E). Guided by the docking/modeling at the AT-1-HMOX1 interface (Figure 6F), we noted that K177 and K179 lie proximal to the predicted binding pocket, suggesting that AT-1 could interfere with ubiquitination at these sites. Indeed, lysine-to-arginine mutants K177R and K179R each showed reduced ubiquitination compared with wild-type (WT) HMOX1, whereas the double mutant (K177/179R) was most resistant (Figure 6G). Functionally, AT-1 failed to stabilize HMOX1 in cells expressing K177/179R, while WT-HMOX1 increased robustly (Figure 6H).

Together, these data support a model in which AT-1 binds HMOX1 and diminishes ubiquitination at K177/K179, thereby stabilizing HMOX1 and promoting ferroptosis in HCC cells.

### Inhibition of HMOX1 blocks the effect of AT-1 on ferroptosis

**Figure 7:**
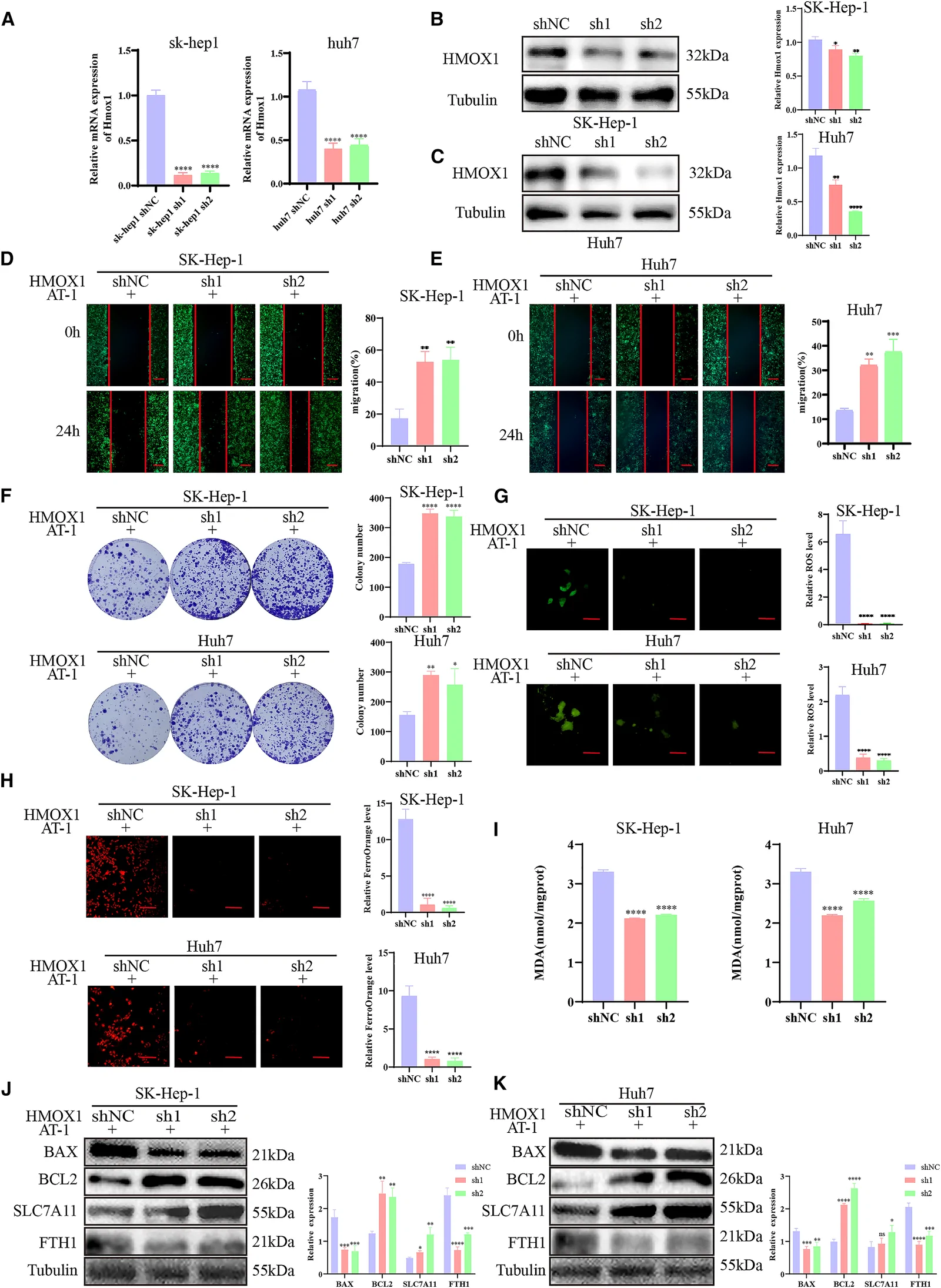
HMOX1 Knockdown Abrogates AT-1-induced ferroptosis and growth inhibition in HCC cells (A–C) Generation of HMOX1-knockdown SK-Hep-1 and Huh7 cells using lentiviral shRNAs (sh1, sh2) with shNC as control. qPCR (A) and immunoblotting (B and C) verify efficient knockdown; Tubulin served as loading control. (D and E) Wound-healing migration after 24 h AT-1 treatment: AT-1 reduces migration in shNC cells but not in shHMOX1 cells; % wound closure quantified. (F) Clonogenic assay: AT-1 diminishes colony formation in shNC cells, with little/no effect in shHMOX1 cells; colonies counted and quantified. (G) ROS measurement by DCFH-DA after 24 h: fluorescence increases with AT-1 in shNC cells but not in shHMOX1 cells. (H) Labile Fe^2+^ staining by FerroOrange after 24 h: AT-1 elevates Fe^2+^ in shNC cells but not in shHMOX1 cells. (I) Lipid peroxidation (MDA) increases upon AT-1 in shNC cells and is abolished by HMOX1 knockdown. (J and K) Western blots of apoptosis/ferroptosis markers: with HMOX1 knockdown, AT-1 no longer upregulates BAX or downregulates BCL2, and the AT-1-induced increase in FTH1 and decrease in SLC7A11 are abolished; densitometric quantifications shown. Significance: ∗*p* < 0.05, ∗∗*p* < 0.01, ∗∗∗*p* < 0.001, ∗∗∗∗*p* < 0.0001. Scale bars, 200 μm in (D) and (E); 100 μm in (G) and (H).

To establish the requirement of HMOX1 for AT-1-mediated ferroptosis, we generated lentiviral shRNA knockdowns in HCC cells (Figures 7A–7C). As anticipated, AT-1 failed to suppress proliferation, invasion, and migration in HMOX1-deficient SK-Hep-1 and Huh7 cells, in contrast to shNC controls (Figures 7D–7F).

At the molecular level, Western blotting showed that the apoptotic response was blunted in HMOX1-knockdown cells: AT-1 did not appreciably increase BAX or decrease BCL-2 relative to controls (Figures 7J and 7K). Pharmacologic inhibition of HMOX1 similarly attenuated AT-1-induced oxidative stress, as evidenced by reduced ROS (Figure 7G), diminished Fe^2+^ accumulation (Figure 7H), and lower MDA levels (Figure 7I). Consistently, the AT-1-driven increase in FTH1 and decrease in SLC7A11 were abolished upon HMOX1 knockdown (Figures 7J and 7K). Collectively, these results indicate that HMOX1 is required for the ferroptotic and anti-proliferative actions of AT-1 *in vitro*.

**Figure 8:**
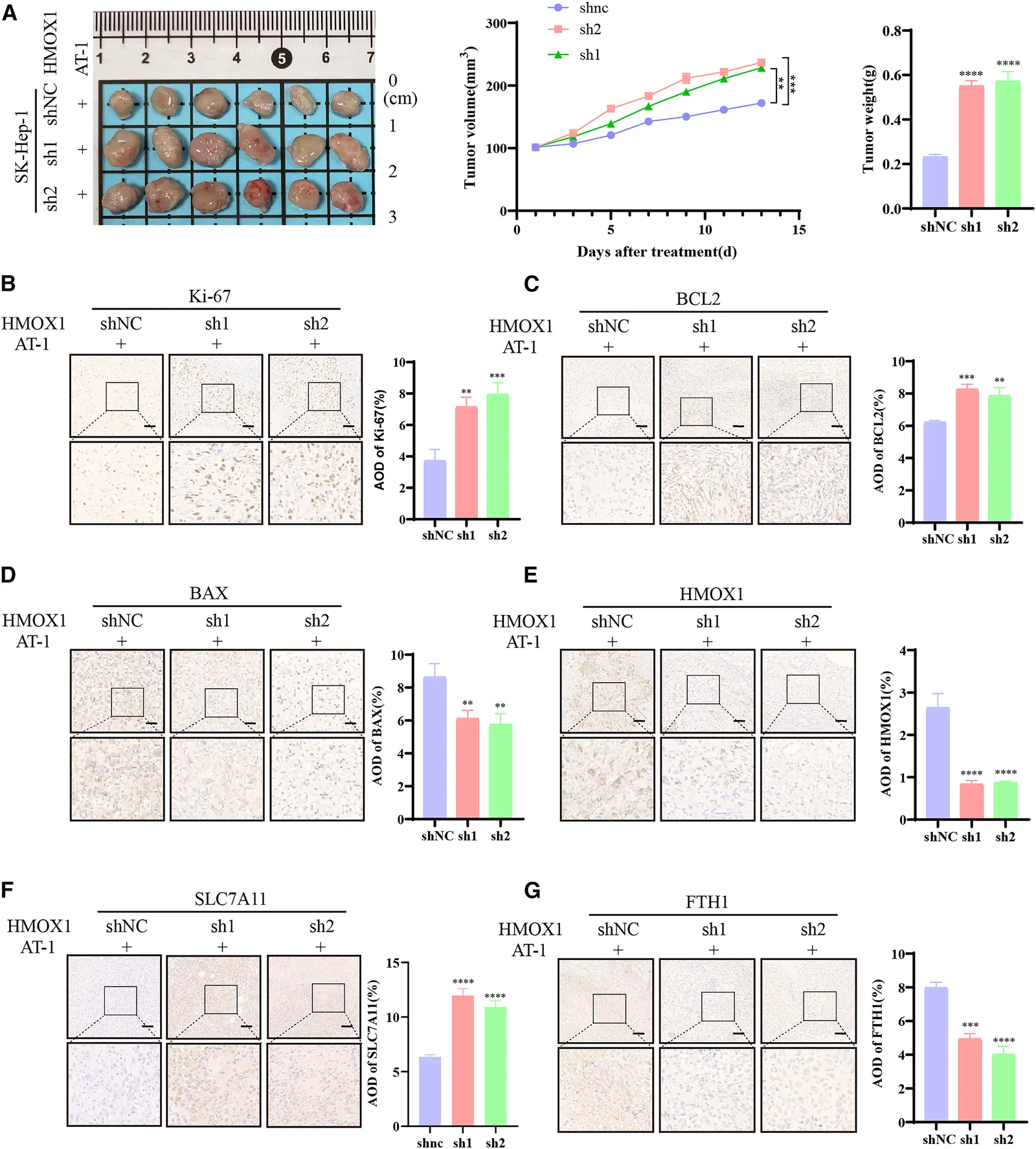
HMOX1 knockdown diminishes the anti-tumor and ferroptosis-related effects of AT-1 *in vivo* (A) Representative photographs of subcutaneous xenografts derived from SK-Hep-1 cells expressing shNC, sh1, or sh2 against HMOX1. Beginning 7 days after inoculation, all cohorts received AT-1 (25 mg/kg, i.p., once daily) for 14 days (*n* = 6 per group). Tumor growth curves (middle; mean ± SEM) and endpoint tumor weights (right) are shown. (B–G) Immunohistochemistry of xenograft sections with representative images (left) and AOD quantification (right): (B) Ki-67 increased, (C) BCL2 increased, (D) BAX decreased, (E) HMOX1 decreased, (F) SLC7A11 increased, and (G) FTH1 decreased in HMOX1-knockdown tumors relative to shNC under AT-1 treatment, indicating loss of AT-1 efficacy when HMOX1 is suppressed. Significance: ∗*p* < 0.05, ∗∗*p* < 0.01, ∗∗∗*p* < 0.001, ∗∗∗∗*p* < 0.0001. Scale bars, 200 μm.

These findings extended *in vivo*. Xenografts established with two HMOX1-knockdown SK-Hep-1 lines exhibited attenuated responses to AT-1: despite treatment, tumors grew faster and were larger at endpoint than shNC controls (Figure 8A). Mice received intraperitoneal injections of AT-1 (25 mg/kg, once daily for 14 days); body weight remained stable, and no abnormal behaviors were observed.

To test the requirement of Hmox1 in an immune-competent setting, we generated stable shHmox1 Hep1-6 cells and established syngeneic tumors in C57BL/6 mice (Figures S2C–S2G). Under the same AT-1 dosing regimen, shHmox1 tumors again failed to be restrained by AT-1, showing faster growth and greater final weights than shNC tumors (Figures S2E–S2G), mirroring the human-cell xenografts.

IHC on tumor sections supported these observations: in HMOX1-knockdown tumors, AT-1 did not downregulate BCL-2 or Ki-67, nor upregulate BAX (Figures 8B–8D). Moreover, HMOX1 knockdown decreased HMOX1 and FTH1 while increasing SLC7A11 relative to shNC controls (Figures 8E–8G). Together, the *in-vitro* and *in-vivo* data demonstrate that HMOX1/Hmox1 is indispensable for AT-1-induced ferroptosis and is pivotal to the anti-tumor efficacy of AT-1 in HCC.

### AT-1 enhances the antitumor efficacy of lenvatinib by stabilizing HMOX1

**Figure 9:**
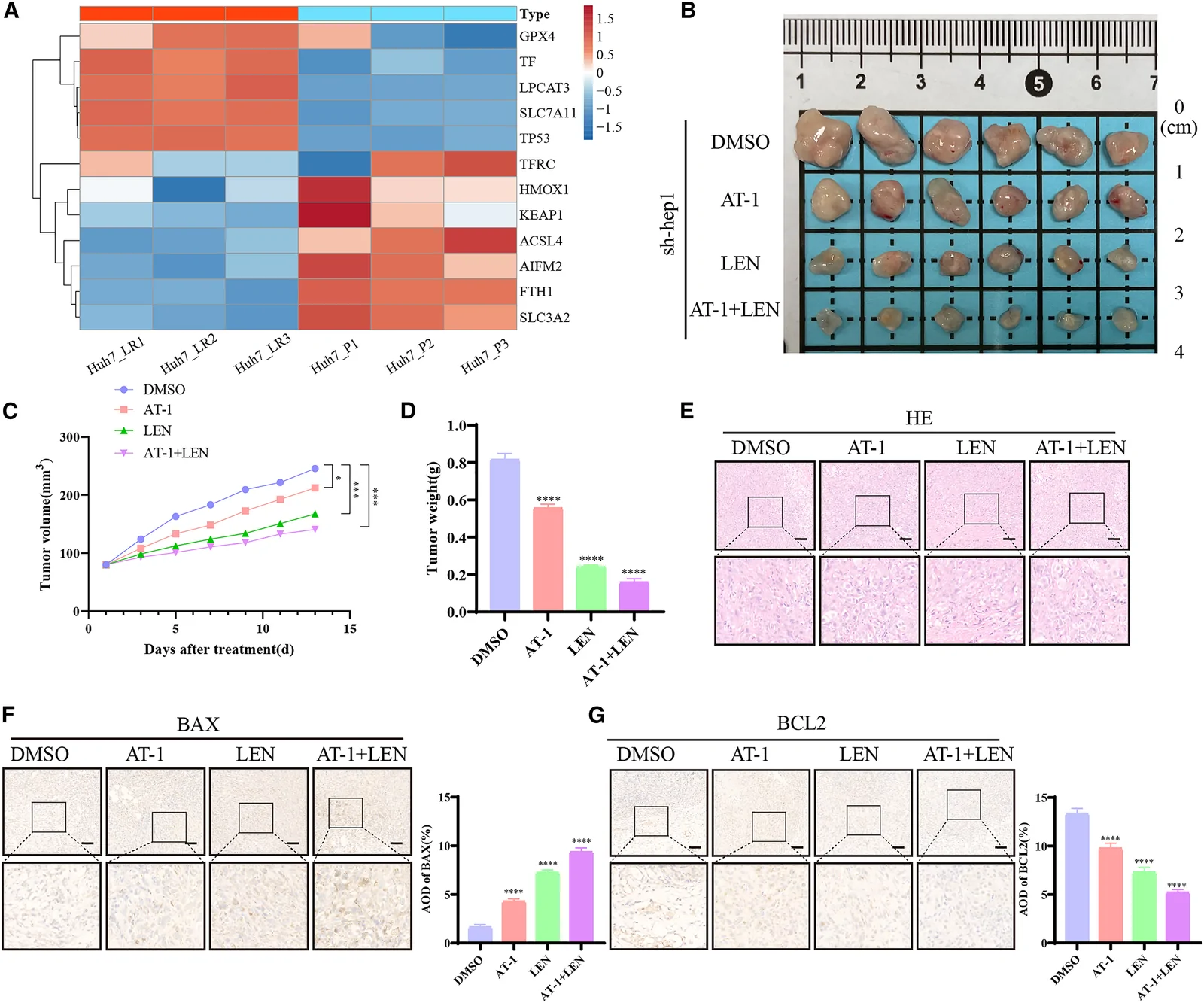
AT-1 enhances the anti-tumor activity of Lenvatinib in HCC xenografts (A) Heatmap of 14 ferroptosis-related genes from the public dataset GSE186191. (B–D) SK-Hep-1 subcutaneous xenografts were treated with vehicle (DMSO), AT-1(25 mg/kg, i.p., once daily), Lenvatinib (LEN; 10 mg/kg, i.p., once daily), or the AT-1(25 mg/kg, i.p.) + LEN (10 mg/kg, i.p., once daily) combination (doses/routes as described in STAR Methods; *n* = 6/group). Representative tumor photographs (B), tumor-growth curves (C; mean ± SEM), and endpoint tumor weights (D; mean ± SEM) are shown. The combination reduced tumor burden more than either monotherapy. (E) H&E staining of tumor sections. (F and G) IHC for BAX and BCL2 with representative images (left) and AOD quantification (right); AT-1 + LEN showed higher BAX and lower BCL2 than single-agent groups. Statistics: two-way ANOVA for growth curves (C) and one-way ANOVA with Tukey/Dunnett for tumor weight and IHC quantifications (D and F–G). Significance: ∗*p* < 0.05, ∗∗*p* < 0.01, ∗∗∗*p* < 0.001, ∗∗∗∗*p* < 0.0001. Scale bars, 100 μm.

Lenvatinib resistance remains a major hurdle in advanced HCC, and emerging evidence implicates ferroptosis tolerance as a contributing mechanism.^34^ Guided by this hypothesis, we analyzed RNA-seq data from GSE186191, comparing three Lenvatinib-resistant Huh7 lines with their parental counterparts. Among 14 core ferroptosis-related genes, HMOX1 was significantly downregulated in resistant cells (Figure 9A), consistent with our finding that AT-1 stabilizes HMOX1 and promotes ferroptosis. We therefore posited that restoring HMOX1 with AT-1 could enhance Lenvatinib efficacy.

In an SK-Hep-1 xenograft model, the AT-1 + Lenvatinib combination outperformed either monotherapy, yielding smaller tumors and lower endpoint weights under the dosing regimen described in STAR Methods (Figures 9B–9D). These *in-vivo* data support the notion that AT-1 augments Lenvatinib activity, consistent with resensitization through restoration of ferroptotic pressure.

Mechanistically aligned tissue readouts were observed by IHC: relative to either agent alone, the combination group exhibited the strongest pro-apoptotic signature—BAX staining increased while BCL-2 decreased (Figures 9E–9G).

Collectively, these results indicate that AT-1, by stabilizing HMOX1 and inducing ferroptosis, enhances—and is consistent with synergistically enhancing—Lenvatinib’s antitumor efficacy in HCC. This combinatorial strategy is promising and warrants further studies in resistant models, with formal synergy quantification and clinical validation.

## Discussion

HCC remains therapeutically challenging, owing to late diagnosis, intratumoral heterogeneity, and acquired drug resistance.^35^ Building on the multi-target, multi-pathway attributes of Traditional Chinese Medicine (TCM),^36^ we identify AT-1—a sesquiterpene lactone from *A. macrocephala*—as a promising anti-HCC agent. Mechanistically, our data support a model in which AT-1 stabilizes HMOX1 to trigger non-canonical, GPX4-independent ferroptosis in HCC.

Across complementary assays, AT-1 produced a coherent ferroptotic signature: labile Fe^2+^ accumulation (FerroOrange), ROS elevation (DCFH-DA), lipid peroxidation (MDA), ferroptosis-like ultrastructure on TEM (mitochondrial shrinkage, condensed membranes, cristae loss), and pharmacologic rescue by ferrostatin-1. Consistent with a GPX4-independent route, GPX4 levels remained stable, while SLC7A11 decreased and FTH1 increased. At the post-translational level, AT-1 prolonged HMOX1 half-life (CHX chase) and reduced HMOX1 ubiquitination; mutating K177/K179 attenuated ubiquitination and abrogated AT-1-mediated stabilization, placing these lysines functionally near the AT-1 interface. *In-silico* docking and MD further supported a stable AT-1-HMOX1 interaction, providing structural rationale for the observed biochemical stabilization. Based on the predicted proximity of AT-1 to the K177/K179 region, we speculate that AT-1 binding may sterically hinder access of the relevant E3 ligase to these lysine residues, or induce a local conformational change that reduces ubiquitin conjugation efficiency.

These mechanistic insights translate into *in-vivo* benefit. In both human SK-Hep-1 xenografts and immune-competent Hep1-6 syngeneic tumors, AT-1 reduced tumor burden without overt toxicity, and tumor IHC mirrored *in-vitro* pathway modulation. Notably, AT-1 enhanced Lenvatinib efficacy *in vivo*. Analysis of Lenvatinib-resistant Huh7 derivatives (GSE186191) showed downregulated HMOX1, aligning with a ferroptosis-tolerance phenotype; AT-1’s capacity to stabilize HMOX1 offers a mechanistic route to restore ferroptotic pressure and augment response. While our data indicate that the combination of AT-1 and Lenvatinib significantly reduces tumor burden compared to either monotherapy, it is important to note that the observed effect appears to be additive rather than synergistic. This additive effect could be quantitatively confirmed by performing a more rigorous analysis, such as calculating the Combination Index (CI) using the Chou-Talalay method or performing a Bliss independence analysis.

Several caveats warrant discussion. First, although we included an immunocompetent model, we did not dissect immune contributions (e.g., lipid peroxidation-driven antigenicity, myeloid reprogramming). Future work should profile the tumor immune microenvironment under AT-1 treatment to explore potential immune modulation. Second, the E3 ligase(s) governing HMOX1 ubiquitination at K177/K179 remain undefined. While our findings suggest that AT-1 stabilizes HMOX1 through inhibition of ubiquitination at these sites, additional studies, such as proteomics (ubiquitin remnant profiling) and mutational scanning, are needed to pinpoint the specific E3 ligase(s) involved. Third, while our *in-silico* evidence supports AT-1 binding to HMOX1, it does not fully substitute for biophysical validation (e.g., ITC, SPR, or DSF) or site-specific ubiquitinomics to directly verify the binding interaction. Fourth, comprehensive ADME/PK (absorption, distribution, metabolism, and excretion/pharmacokinetics), off-target assessment, and repeat-dose toxicology are necessary to de-risk translation to clinical settings. Finally, while AT-1 constrained proliferation and motility *in vitro*, metastatic suppression *in vivo* was not directly tested. This should be addressed in orthotopic or dissemination models to fully evaluate the therapeutic potential of AT-1.

HMOX1 is traditionally recognized as a cytoprotective enzyme due to its role in maintaining iron homeostasis and protecting against oxidative stress.^30^ However, under certain stress conditions, particularly when there is excessive iron accumulation, HMOX1 can become a “double-edged sword.” Excessive accumulation of Fe^2+^ can trigger ferroptosis, a form of iron-dependent cell death characterized by lipid peroxidation and mitochondrial dysfunction.^37^ In our study, we observed that AT-1 stabilizes HMOX1, which leads to increased Fe^2+^ accumulation, lipid peroxidation, and ferroptosis. This suggests that while HMOX1 generally acts as a protective enzyme, its upregulation under specific conditions, such as iron overload induced by AT-1, contributes to cell death rather than protection.

In summary, AT-1 stabilizes HMOX1 to induce non-canonical ferroptosis and exerts antitumor activity in HCC, while enhancing Lenvatinib efficacy *in vivo*. By bridging TCM-derived phytochemistry and modern ferroptosis biology, our study nominates HMOX1 as a tractable node for combination therapy in HCC and positions AT-1 as a candidate ferroptosis-enabling agent. Future studies should (1) define the HMOX1 ubiquitination axis and its E3 ligase(s), (2) formally establish drug synergy and optimal scheduling with Lenvatinib, (3) expand to immune-mechanistic readouts, and (4) progress PK/toxicology toward early-phase clinical evaluation, particularly in Lenvatinib-refractory disease.

### Limitations of the study

This study has several key limitations. First, AT-1’s regulatory effects on the HCC tumor immune microenvironment were not explored. Second, the specific E3 ligases mediating HMOX1 K177/K179 ubiquitination remain unidentified. Moreover, comprehensive ADME/PK profiles, systematic toxicological data, and *in-vivo* evidence for AT-1’s anti-metastatic activity are still lacking, requiring further preclinical verification.

## Resource availability

### Lead contact

Requests for further information and resources should be directed to and will be fulfilled by the lead contact, Dousheng Bai (drbaidousheng@yzu.edu.cn).

### Materials availability

This study did not generate new unique reagents.

### Data and code availability

• A publicly available RNA-seq dataset for Lenvatinib-resistant HCC cells was retrieved from the NCBI GEO repository under accession number GSE186191, a Cell Press-approved transcriptomics database. All data reported in this paper will be shared by the lead contact upon request.
• This paper does not report original code.
• Any additional information required to reanalyze the data reported in this paper is available from the lead contact upon request.

## Acknowledgments

This work was supported by the 10.13039/501100001809National Natural Science Foundation of China (82373034, 82503422, and 82203716); the “13th Five-Year Plan” Science and Education Strong Health Project Innovation Team of Yangzhou (LJRC20181 and YZCXTD201801); the Provincial-Level Discipline Leader of the NJPH (DTRA202214); the Cross-Cooperation Special Projects of the NJPH (YJCHZ-2021-08); the Beijing iGanDan Foundation (GDXZ-08-19); the Yangzhou Basic Research Program (Joint Special Project) (2024-3-02); the Health Research Major Project in Jiangsu Province (K2023002); and the Postgraduate Research and Practice Innovation Program of Jiangsu Province (KYCX23_3617).

## Author contributions

Conceptualization, methodology, investigation, formal analysis, and writing – original draft, Y.M. and J.C.; investigation and data curation, R.P., D.T., and J.Z.; conceptualization, methodology, and supervision, S.F., B.S., A.J., J.Z., and S.J.; supervision, writing – review and editing, and final approval, G.J., C.Z., J.C., and D.B. All authors read and approved the final manuscript.

## Declaration of interests

The authors declare no competing interests.

## STAR★Methods

### Key resources table

**Table:** 

REAGENT or RESOURCE	SOURCE	IDENTIFIER
**Antibodies**		

Mouse anti-HMOX1	Santa Cruz	Cat# sc-136960
Mouse anti-GPX4	Santa Cruz	Cat# sc-166570
Mouse anti-FTH1	Santa Cruz	Cat# sc-376594
Rabbit anti-SLC7A11	Biodragon	Cat# RM5446
Rabbit anti-Bax	Zenbio	Cat# R380709
Rabbit anti-Bcl2	Zenbio	Cat# R381702
Rabbit anti-PRKCA	Zenbio	Cat# R381631
Rabbit anti-CTSB	Zenbio	Cat# R381757
Rabbit anti-TP53	Zenbio	Cat# R345567
Rabbit anti-Vinculin	Proteintech	Cat# 26520-1-AP
Rabbit anti-Tubulin	Proteintech	Cat# 10094-1-AP
Mouse anti-Ubiquitin	Santa Cruz	Cat# sc-8017
Rabbit anti-FLAG	Proteintech	Cat# 20543-1-AP

**Bacterial and virus strains**		

short hairpin RNA (shRNA) expression vectors	Guangzhou RiboBio Biotechnology Co., Ltd. (Guangzhou, China)	N/A
overexpression plasmids	Guangzhou RiboBio Biotechnology Co., Ltd. (Guangzhou, China)	N/A
negative control vectors	Guangzhou RiboBio Biotechnology Co., Ltd. (Guangzhou, China)	N/A

**Chemicals, peptides, and recombinant proteins**		

Atractylenolide I (AT-1)	MedChemExpress	Cat# HY-N0201

**Critical commercial assays**		

RNA Sequencing	Shanghai Meiji Biomedical Technology Co., Ltd.	N/A

**Deposited data**		

HCC RNA-seq dataset	GEO Database	GSE186191

**Experimental models: Cell lines**		

Huh7 (Human HCC)	Cell Bank of Chinese Academy of Sciences, Shanghai	STR-authenticated, Mycoplasma-free
SK-Hep-1 (Human HCC)	Cell Bank of Chinese Academy of Sciences, Shanghai	STR-authenticated, Mycoplasma-free
Hep1-6 (Mouse HCC)	Cell Bank of Chinese Academy of Sciences, Shanghai	STR-authenticated, Mycoplasma-free

**Experimental models: Organisms/strains**		

BALB/c nude mice	Yangzhou University Translational Medical Center	N/A
C57BL/6 mice	Yangzhou University Translational Medical Center	N/A

**Oligonucleotides**		

HMOX1-F	Sangon	GCCAGTGCCACCAAGTTCAAG
HMOX1-R	Sangon	GATGTTGAGCAGGAACGCAGTC
β-actin-F	Sangon	CATGTACGTTGCTATCCAGGC
β-actin-R	Sangon	CTCCTTAATGTCACGCACGAT

**Software and algorithms**		

ImageJ	NIH	v1.54
FlowJo	Tree Star	v7.6
GraphPad Prism	GraphPad Software	v9.0
ChemOffice	PerkinElmer	v2022
AutoDock Vina	Open-source	v1.2.6
PyMOL	Schrödinger	v3.0.3
GROMACS	GROMACSOpen-source	v2022
SPSS Statistics	SPSS Inc.	v21.0

### Experimental model and study participant details

#### Human HCC cell lines

Two human hepatocellular carcinoma cell lines, Huh7 and SK-Hep-1, were purchased from the Cell Bank of the Chinese Academy of Sciences (Shanghai, China). Full cell authentication was performed via short tandem repeat (STR) profiling upon receipt, and mycoplasma contamination was excluded using PCR-based detection. The biological sex of the original human tissue donors for both cell lines was not disclosed by the cell bank, which restricts sex-stratified cellular functional analyses.

All cells were cultured under standardized humidified incubator conditions (37 °C, 5% CO_2_). Complete culture medium consisted of high-glucose DMEM supplemented with 10% fetal bovine serum (FBS) and 1% penicillin–streptomycin mixture.

RRID identifiers for Huh7 and SK-Hep-1 were recorded and listed in the key resources table (KRT).

#### Vertebrate laboratory mice

Four-week-old male BALB/c nude mice and 6–8-week-old male C57BL/6 mice were sourced from the Translational Medical Center of Yangzhou University. All animals were maintained in a standardized specific-pathogen-free (SPF) animal facility with a 12 h light/dark cycle, constant ambient temperature (22 ± 2 °C), and free access to standard rodent chow and sterile drinking water. Mice were group-housed with littermates before subcutaneous tumor implantation.

All *in vivo* vertebrate animal experiments received ethical approval from the Laboratory Animal Ethics Committee of Yangzhou University (approval ID: yzu-lcyxy-n030). Experimental operations followed institutional laboratory animal care guidelines and the ARRIVE reporting framework. Mice were randomly allocated to treatment or vehicle groups via computer-generated random sequences after tumor formation was palpable. All mice were humanely euthanized 24 h after the final drug administration.

### Method details

#### Lentiviral vector construction and stable cell selection

Short hairpin RNA (shRNA) knockdown plasmids, target overexpression vectors, and corresponding negative control lentiviral backbones were purchased from Guangzhou RiboBio Biotechnology Co., Ltd. HCC cells were transduced with lentivirus in complete DMEM medium. After 72 h continuous puromycin selection (2 μg/mL), surviving cells were expanded to generate homogeneous stably transduced cell populations for downstream functional assays.

#### Atractylenolide I compound preparation and vehicle control rules

Atractylenolide I (AT-1) powder was dissolved in pure DMSO to prepare a 100 mM stock solution, split into single-use aliquots, and stored at −20 °C under light protection. Fresh working concentrations were diluted in complete culture medium immediately before each experiment. For all *in vitro* assays, the final DMSO concentration across all treatment groups was capped at 0.1% (v/v); vehicle control groups received an equivalent volume of DMSO-matched medium. Full *in vivo* AT-1 administration protocols are described in the subcutaneous xenograft model subsection.

#### Colony formation proliferation assay

Huh7 and SK-Hep-1 cells were seeded into 6-well plates at low densities calibrated to yield 50–200 distinct colonies per well. Cells were cultured continuously for 14 days, with full medium replenishment every 2–3 days. At the experimental endpoint, monolayers were fixed in 4% paraformaldehyde for 30 min at room temperature, stained with 0.1% crystal violet solution for 30 min, rinsed with distilled water, and air-dried fully.

Colonies containing ≥50 individual cells were captured via brightfield microscopy. Colony count and total colony area were quantified using ImageJ software under blinded analysis to eliminate observer bias. Each experimental condition included 3 technical replicate wells, and the entire assay was independently repeated a minimum of 3 biological times.

#### CCK-8 cell viability and dose-response testing

HCC cells were seeded into 96-well plates at optimized densities (2–4 × 10^3^ cells per 100 μL medium) and incubated overnight at 37 °C to achieve full cellular adherence. Cells were exposed to serially diluted AT-1 for 24 h, with DMSO vehicle controls included for every concentration gradient.

Post-treatment, 10 μL CCK-8 reagent was added to each well, and plates were incubated at 37 °C in darkness for 1–2 h (incubation duration adjusted to maintain absorbance within linear detection range). Optical density at 450 nm was measured using a microplate reader; blank wells containing medium + CCK-8 without cells were used to subtract background absorbance.

Cell viability was calculated by the formula: Viability (%) = 100 × (ODtreated − ODblank)/(ODvehicle − ODblank). Dose–response curves were normalized to vehicle control values and fitted to a four-parameter logistic regression model in GraphPad Prism to calculate IC_50_ half-maximal inhibitory concentrations. Each treatment condition contained ≥3 technical replicates, with 3 independent biological repetitions of the full dose series.

#### Wound healing cell migration assay

HCC cells were cultured in 6-well plates until monolayers reached ∼80% confluence. A sterile 200 μL pipette tip was used to create uniform linear scratches across the cell layer; detached floating cells were washed twice with pre-warmed PBS. Culture medium was replaced with low-serum DMEM (0.5–1% FBS) supplemented with AT-1 or matched DMSO vehicle (0.1% final DMSO).

Plates were returned to a 37 °C, 5% CO_2_ incubator. Identical scratch regions were imaged at 0 h (immediately post-scratch) and 24 h post-treatment; plate bottom marks guided consistent field selection during imaging. The cell-free wound area at each time point was quantified via ImageJ threshold segmentation under blinded analysis. Wound closure rate was defined as: Closure (%) = 100 × (A_0_ − A_24_)/A_0_, where A_0_ = wound area at 0 h, A_24_ = wound area at 24 h. A minimum of 5 independent microscopic fields were quantified per replicate well, with 3 technical replicates and ≥3 independent biological repeats per condition.

#### Annexin V-FITC/PI flow cytometry apoptosis detection

SK-Hep-1 and Huh7 cells were treated with gradient AT-1 concentrations or DMSO vehicle for 24 h. Cells were harvested via trypsinization, washed twice with ice-cold PBS, and resuspended in Ca^2+^-containing Annexin V binding buffer following the kit manufacturer’s standard protocol.

Per 100–200 μL cell suspension (1 × 10^5^ total cells), Annexin V-FITC and propidium iodide (PI) staining reagents were added. Samples were incubated for 10–15 min at room temperature in complete darkness, then diluted with additional binding buffer before flow acquisition. Unstained control, single-stain (FITC-only, PI-only), and fluorescence-minus-one (FMO) control samples were run alongside treated groups to calibrate instrument voltage and spectral compensation.

A minimum of 10,000 singlet cellular events were collected per sample within 1 h of staining on a flow cytometer with linear FSC/SSC and logarithmic fluorescence scaling. Raw data were analyzed using FlowJo v7.6 software. Cellular debris was excluded via FSC-A/SSC-A gating, and cell doublets were removed using FSC-H vs. FSC-A contour plots. Dot plot quadrants were defined to separate viable, early apoptotic, late apoptotic/necrotic, and necrotic populations. Total apoptosis percentage was calculated as the combined fraction of early + late apoptotic singlet cells. All flow data analysis was performed blinded to treatment group allocation; each condition contained ≥3 technical replicates and ≥3 independent biological experiments.

#### Immunohistochemical staining of paraffin tissue sections

Formalin-fixed HCC tissue specimens were embedded in paraffin wax and cut into continuous 4 μm thick sections. Slides underwent full deparaffinization in xylene and serial rehydration through graded ethanol solutions. Endogenous peroxidase activity was quenched with 3% H_2_O_2_ for 10 min at room temperature. Heat-induced epitope retrieval was performed using either 10 mM citrate buffer (pH 6.0) or 1 mM EDTA buffer (pH 8.0–9.0), selected according to the primary antibody manufacturer’s specifications.

Non-specific binding was blocked with 5% BSA solution for 30 min at room temperature. Sections were incubated with diluted primary antibodies overnight at 4 °C, followed by HRP-conjugated secondary antibodies for 1 h at room temperature. DAB chromogenic substrate was applied to visualize antigen signals, and cell nuclei were counterstained with hematoxylin. Negative control slides were processed in parallel with primary antibody omitted to exclude non-specific background staining. A commercial IHC detection kit (Absin, Shanghai) was used for all staining procedures following the enclosed manufacturer protocol.

#### Western blot and RT-qPCR gene expression quantification

WB and RT–qPCR were performed essentially as described previously,^38^ with the following details. For Western blot (WB): Cells or tumor tissue samples were lysed on ice in RIPA buffer supplemented with protease and phosphatase inhibitor cocktails. Total protein concentration was quantified via BCA colorimetric assay. Equal mass protein samples were separated by SDS–PAGE and electrotransferred onto PVDF membranes. Membranes were blocked with 5% non-fat milk or BSA for 1 h at room temperature, incubated with primary antibodies overnight at 4°C, and probed with HRP-linked secondary antibodies for 1 h at room temperature. Target protein bands were visualized using ECL chemiluminescent substrate and captured with a digital chemiluminescence imager. Band gray intensity was quantified via ImageJ software and normalized to constitutively expressed loading control proteins for relative expression calculation.

For RT-qPCR: Total cellular RNA was extracted using TRIzol reagent, and complementary DNA (cDNA) was reverse-transcribed using a commercial reverse transcription kit per manufacturer instructions. Quantitative PCR amplification was performed with SYBR Green fluorescent dye on a real-time thermal cycler. Melting curve analysis confirmed single specific amplification products for all primer pairs. Relative mRNA expression levels were calculated via the 2^∧^−ΔΔCt method with GAPDH as the internal housekeeping reference gene. All primer oligonucleotide sequences are provided in key resources table. Each experimental group contained 3 independent biological replicates, with technical triplicate qPCR reactions for every RNA sample.

#### RNA-seq library construction and raw data acquisition

Huh7 cells were split into two experimental cohorts: DMSO vehicle control and 100 μM AT-1 treatment group. After 24 h compound exposure, cells were digested with trypsin, pelleted via centrifugation, and washed three times with ice-cold PBS. Purified cell pellets were shipped to Shanghai Meiji Biomedical Technology Co., Ltd. for total RNA extraction, library construction, paired-end RNA sequencing, and downstream bioinformatic processing.

#### MDA lipid peroxidation quantitative colorimetric assay

Cellular malondialdehyde (MDA) levels were measured using a commercial detection kit (Beyotime, catalog S0131M). HCC cells were seeded into 6-well plates, cultured overnight for adherence, and treated with serially diluted AT-1 or DMSO vehicle for 24 h. Post-treatment, cells were washed with ice-cold PBS, scraped, and fully lysed on ice. Clarified supernatant total protein content was determined via BCA assay for normalization.

Lysate aliquots were mixed with kit-supplied MDA working solution and heated to 95 °C for 15 min to generate TBA-MDA chromogenic adducts. Reaction mixtures were cooled to room temperature and centrifuged to remove insoluble precipitates. Supernatants were transferred to clear 96-well plates, and absorbance at 532 nm was measured against a reagent-only blank control. Absolute MDA concentration was interpolated from a standard curve generated with kit-provided MDA standards and normalized to total cellular protein to report nmol MDA per mg protein. Each condition contained 3 technical replicate wells, and the full assay was independently repeated ≥3 times.

#### DCFH-DA intracellular ROS fluorescence imaging

HCC cells were seeded at 1 × 10^6^ cells per 6-well plate and cultured overnight to adhere. Cells were treated with AT-1 concentration gradients or DMSO vehicle for 24 h. Culture medium was removed, and cells were incubated with 10 μM DCFH-DA fluorescent probe (diluted 1:1000 in serum-free DMEM) for 20 min at 37 °C in complete darkness. Unbound extracellular probe was washed away three times with serum-free medium, and cells were equilibrated in phenol-red-free HBSS imaging buffer. Fluorescence micrographs were captured on an inverted fluorescence microscope with identical fixed exposure parameters across all experimental groups to enable direct signal comparison. Each treatment group contained ≥3 technical replicate wells, with ≥3 independent biological imaging experiments.

#### FerroOrange intracellular ferrous ion (Fe^2+^) staining

HCC cells were seeded onto 24-well culture plates and incubated overnight for adherence. Cells were exposed to AT-1 or matched DMSO vehicle for 24 h. After two PBS washes, cells were incubated with 1 μM FerroOrange fluorescent probe (Dojindo, catalog F374; diluted in phenol-red-free HBSS) for 30 min at 37 °C in darkness. Excess probe was removed via three sequential washes with imaging buffer. TRITC channel fluorescence images were acquired with standardized microscope exposure settings across all groups; nuclei were optionally counterstained with Hoechst 33342 to normalize for cell density differences. Each condition included ≥3 technical replicates, and staining experiments were repeated independently at least three times.

#### Transmission electron microscopy (TEM) ultrastructure imaging

HCC cell pellets were fixed in 2.5% glutaraldehyde (diluted in 0.1 M phosphate buffer, pH 7.4) for 12 h at 4 °C. Samples were thoroughly rinsed with buffer solution and post-fixed in 2% osmium tetroxide for 1 h at 4 °C. After washing, cell pellets underwent *en bloc* staining with 1% aqueous uranyl acetate (dark conditions), followed by serial dehydration in graded ethanol (50%, 70%, 90%, 95%, 100%). Dehydrated samples were infiltrated and embedded in Eponate 12 resin per manufacturer protocols, then polymerized at 60 °C for 24–48 h. Ultrathin sections (70–90 nm thickness) were cut using an ultramicrotome and mounted onto copper grids. Grids were sequentially post-stained with uranyl acetate (dark incubation) and lead citrate (CO_2_-free environment). Cellular ultrastructure images were captured on a Tecnai G2 Spirit TEM operating at 120 kV accelerating voltage. For every treatment group, ≥5–10 distinct microscopic fields were imaged from a minimum of two independent resin blocks under blinded group labeling.

#### HMOX1 ubiquitination co-IP and cycloheximide chase assay

For ubiquitination immunoprecipitation (IP): SK-Hep-1 cells were treated with AT-1 or DMSO vehicle for 24 h; proteasome inhibitor MG132 (10 μM) was added for the final 4 h of incubation to enrich ubiquitinated protein substrates before cell harvest. Cells were lysed on ice in denaturing IP buffer, heated to 95 °C for 5 min, then diluted 10-fold with non-denaturing buffer to reduce SDS concentration to 0.1%. Clarified whole-cell lysates were incubated with anti-HMOX1 primary antibody overnight at 4 °C; antibody-protein complexes were captured with Protein A/G magnetic agarose beads over 1–2 h at 4 °C. Beads were washed 3–5 times with cold IP wash buffer, and bound proteins were eluted in 2× Laemmli loading buffer at 95 °C for 5 min. Eluates were separated via SDS-PAGE and immunoblotted with anti-ubiquitin antibody to detect HMOX1 ubiquitination; total HMOX1 input levels were re-probed to validate IP efficiency.

For cycloheximide (CHX) protein stability chase: SK-Hep-1 cells were co-treated with 50 μg/mL CHX (Selleck, catalog S7418) to block *de novo* protein synthesis, with or without AT-1 co-administration. Cells were harvested at predefined sequential time points, and total HMOX1 protein abundance was detected via Western blot as described above. Band intensity was quantified with ImageJ software and normalized to housekeeping protein loading controls to calculate relative HMOX1 half-life.

#### Small molecule–protein molecular docking workflow

Two-dimensional structural files of AT-1 were retrieved from the PubChem database and converted to energy-minimized 3D conformations using ChemOffice software (PerkinElmer). Processed ligand structures (.mol2 format) were optimized to remove steric strain prior to docking. High-resolution target protein crystal structures were downloaded from the RCSB PDB repository. In PyMOL software, non-essential water molecules, buffer ions, and irrelevant heteroatoms were deleted; catalytic cofactors critical to protein structure were retained.

Protein receptor and AT-1 ligand files were prepared in AutoDockTools (ADT) v1.5.6: polar hydrogens were added, Gasteiger partial charges were assigned, and all structures were exported to PDBQT format. Molecular docking simulations were executed with AutoDock Vina software with standardized parameters: exhaustiveness = 8–16, num_modes = 20, energy_range = 4, fixed random seed for full result reproducibility. Top-ranked ligand binding poses were filtered by Vina binding energy score and structural cluster convergence, then manually inspected for hydrogen bonds, π–π/π–cation interactions, and steric compatibility. Two-dimensional interaction heatmaps and three-dimensional binding pocket visualizations were generated in PyMOL and Discovery Studio 2019. Docking affinity scores are reported in kcal·mol^−1^; scores < −5.0 kcal mol^−1^ indicate moderate binding affinity, while scores < −7.0 kcal mol^−1^ reflect strong predicted molecular interaction, used qualitatively to prioritize candidate binding conformations.

#### All-atom molecular dynamics (MD) simulation (100 ns)

100 ns all-atom molecular dynamics simulations were performed for the AT-1–target protein complex using GROMACS 2022 software. Protein atomic interactions were parameterized with the CHARMM36 force field; AT-1 ligand atomic charges and bonding parameters were generated via GAFF2 with AM1-BCC charge calculation, converted to GROMACS-compatible topology files using acpype/Antechamber.

Each protein–ligand complex was solvated in a dodecahedral TIP3P water box with a minimum 1.2 nm solvent buffer between all protein atoms and box boundaries. System ionic strength was adjusted to 0.15 M NaCl, and net molecular charge was neutralized with counterions. Energy minimization was performed via steepest descent algorithm until maximum atomic force Fmax <1000 kJ mol^−1^ nm^−1^. Short-range van der Waals and electrostatic interactions used a 1.0 nm real-space cutoff with Verlet neighbor list scheme; long-range electrostatic forces were calculated via particle-mesh Ewald (PME) summation. All hydrogen-containing chemical bonds were constrained using the LINCS algorithm, enabling a 2 fs simulation integration timestep.

Two sequential equilibration phases were run: 100 ps NVT ensemble equilibration at 310 K with velocity-rescale thermostat (τ_t_ = 0.1 ps), followed by 100 ps NPT ensemble equilibration at 1 bar with Parrinello–Rahman barostat (τ_p_ = 2.0 ps, compressibility = 4.5 × 10^−5^ bar^−1^). Heavy protein atoms were restrained with positional force constants (1000 kJ mol^−1^ nm^−2^) during equilibration to preserve native protein fold. Unrestrained production MD simulation proceeded for 100 ns at 310 K and 1 bar with periodic boundary conditions applied across all three spatial axes. Atomic coordinate and system energy snapshots were saved every 10 ps for post-simulation analysis.

Trajectory analysis (root-mean-square deviation (RMSD), root-mean-square fluctuation (RMSF), radius of gyration, intramolecular hydrogen bond count, ligand–residue contact distances) was completed using built-in GROMACS analysis tools. Complex structural stability was assessed via plateaued RMSD values and persistent ligand–protein intermolecular contacts. One independent 100 ns trajectory was generated for the AT-1–protein complex unless otherwise specified.

#### Public GEO RNA-seq dataset bioinformatic analysis

Public HCC cell RNA-seq raw data were downloaded from the NCBI Gene Expression Omnibus (GEO) database under accession ID GSE186191, containing six independent cell sample profiles. Differentially expressed mRNA transcripts (DEGs) were screened using dual thresholds: |log_2_(fold change)| > 1 and adjusted *p*-value <0.05. Heatmaps visualizing ferroptosis-related gene expression patterns were plotted in R language using the ‘pheatmap’ and ‘ggplot2’ packages.

#### Subcutaneous HCC xenograft mouse tumor model

Four-week-old male BALB/c nude mice and 6–8-week-old male C57BL/6 mice were used for subcutaneous tumor implantation. SK-Hep-1 human HCC cells (for nude mice) or Hep1-6 mouse HCC cells (for C57BL/6 mice) were resuspended in sterile PBS at a density of 5 × 10^6^ cells per 100 μL suspension, then injected subcutaneously into the dorsal flank of each mouse (*n* = 6 animals per experimental group).

Tumor length (L), width (W), and whole-body mouse weight were measured every 3 days using a digital caliper by an investigator blinded to treatment group allocation. Tumor volume was calculated using the standard ellipsoid formula: V = (L × W^2^)/2.

Tumors became palpable approximately 7 days post-cell injection; mice were randomly assigned to AT-1 treatment or vehicle groups via computer-generated randomization sequences. The AT-1 treatment cohort received daily intraperitoneal (i.p.) injections of 25 mg/kg AT-1 for 14 consecutive days; control mice received an equivalent volume of DMSO-containing vehicle solution administered via matching i.p. route and schedule. All mice were euthanized 24 h after the final injection. Excised tumor tissues were weighed, fixed, and preserved for IHC, Western blot, and RNA quantification downstream analyses. All *in vivo* procedures complied with Yangzhou University institutional animal care regulations and ARRIVE reporting standards.

### Quantification and statistical analysis

All statistical tests, software, replicate definitions, significance thresholds, and blinding protocols are fully detailed below. All statistical parameters are reported within figure legends and the main Results text.

#### Statistical software

All quantitative analyses were performed using SPSS Statistics v21.0 (SPSS Inc., Chicago, USA).

#### Group comparison tests

Unpaired two-tailed Student’s *t* test was used for pairwise comparisons between two independent groups; paired two-sample Wilcoxon rank-sum test was applied for non-normally distributed paired datasets. For comparisons across three or more experimental groups, one-way analysis of variance (one-way ANOVA) followed by post-hoc pairwise multiple comparison tests was implemented.

#### Data distribution and exclusion criteria

No formal normality or homogeneity of variance testing was performed prior to statistical modeling; no outlier data points were manually excluded from any dataset. All raw quantitative measurements were retained for analysis.

#### Replicate definition

• Cellular functional assays (colony formation, CCK-8, wound healing, flow cytometry, fluorescence staining): n = independent biological cell culture replicates (minimum 3 per assay); each biological replicate contained 3 technical replicate wells.
• *In vivo* xenograft mouse experiments: n = individual mice per treatment group (n = 6).
• TEM, immunofluorescence, IHC imaging: n = independent biological cell/tissue samples, with ≥5 microscopic fields quantified per sample under blinded analysis.
• Molecular docking & MD simulation: Single independent computational trajectory per protein–ligand complex.

#### Data presentation and dispersion metrics

All quantitative data are displayed as mean ± standard error of the mean (SEM).

#### Significance cutoff and asterisk definitions

A *p*-value <0.05 was defined as the threshold for statistically significant differences between groups. Asterisk markers used in all figures correspond to *p* values as follows: ∗*p* < 0.05, ∗∗*p* < 0.01, ∗∗∗*p* < 0.001, ∗∗∗∗*p* < 0.0001.

#### Blinding implementation

All manual quantitative readouts (tumor volume measurement, colony counting, wound area calculation, TEM image scoring, immunofluorescence signal quantification) were completed by researchers blinded to sample treatment group identity to eliminate observer bias.

### Additional resources

This study did not generate dedicated online protocols, data repositories, or clinical trial registrations; this section is omitted per journal guidelines.
