# Supplementary material for: Atractylenolide I stabilizes HMOX1 to induce ferroptosis and enhance lenvatinib efficacy in hepatocellular carcinoma
Source: iScience. 2026 Aug 21;29(9):117259. doi: 10.1016/j.isci.2026.117259 (PMC13524718; doi:10.1016/j.isci.2026.117259)
Supplement: Supplementary file 1 [file mmc1.pdf]

iScience, Volume *29*

Supplemental information

Atractylenolide I stabilizes HMOX1 to induce ferroptosis and enhance lenvatinib efficacy in hepatocellular carcinoma

Yangyang Miao, Shengjie Jin, Rui Peng, Daoyuan Tu, Jiahao Zhang, Songsong Fan, Bingbing Su, Anyang Jiang, Jihu Zheng, Guoqing Jiang, Chi Zhang, Jun Cao, and Dousheng Bai

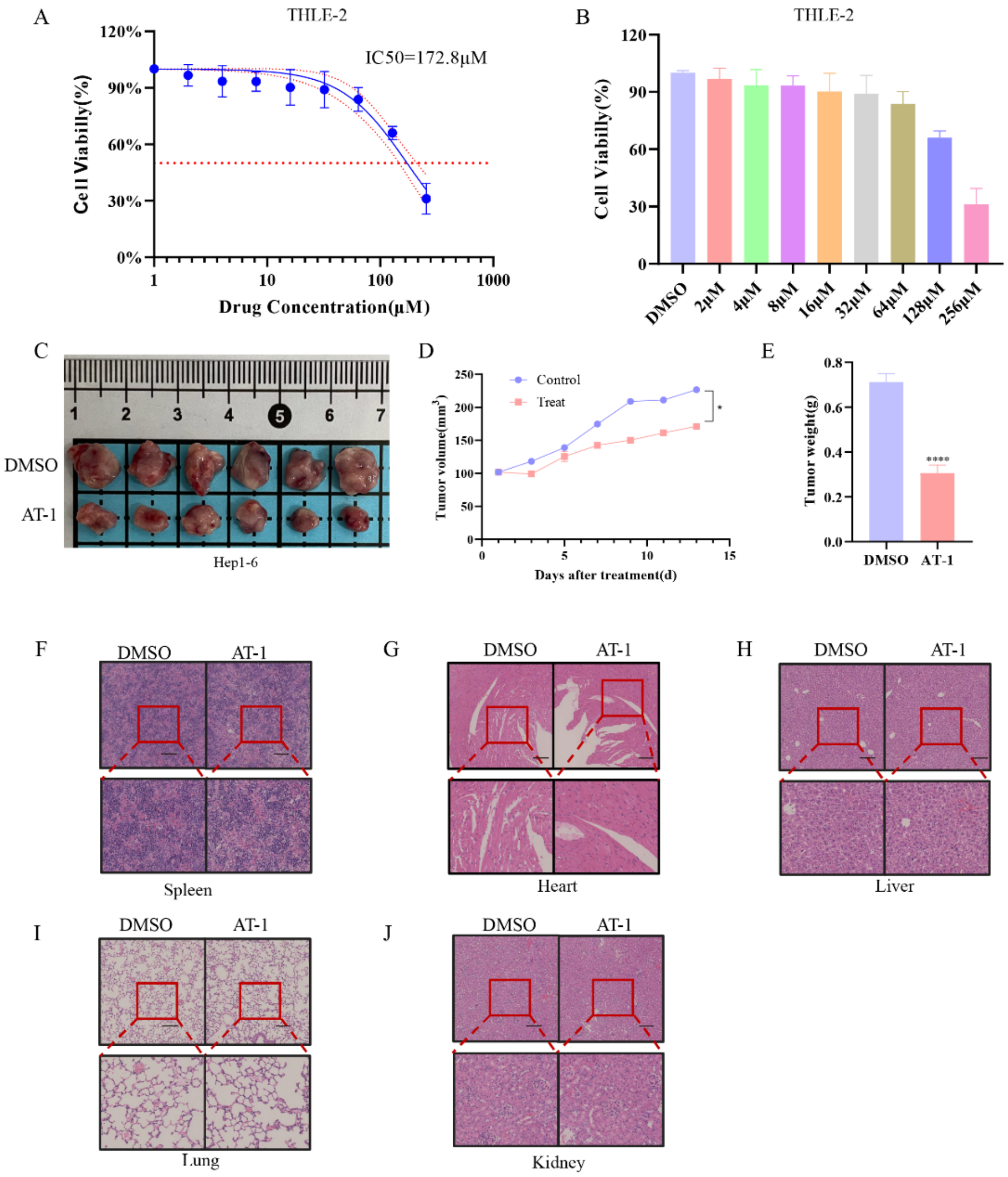

Supplementary Figure 1. cytotoxicity of AT-1 in normal hepatocytes and in-vivo safety assessment

(A) Dose–response curve of THLE-2 cells treated with AT-1 for 24 h; viability normalized to DMSO and fitted with a 4-parameter logistic model, yielding $IC_{50} \approx 172.8$ μM. (B) Bar plot of THLE-2 viability at the indicated AT-1 concentrations (24 h). (C–E) Representative photographs of excised tumors (C), tumor-growth curves (D; mean ± SEM), and endpoint tumor weights (E; mean ± SEM) from Hep1-6 tumors treated with DMSO or AT-1 (25 mg/kg, i.p., once daily for 14 days); n = 6 mice per group. (F-J). Hematoxylin–eosin (H&E) staining of spleen (F), heart (G), liver (H), lung (I), and kidney (J) collected at endpoint from DMSO- and AT-1–treated mice; no overt histopathological alterations were observed. Data are presented as mean ± SEM. Statistics: two-way ANOVA for tumor-growth curves (D); two-tailed unpaired t-tests

for pairwise comparisons (E and B). Significance: * $p < 0.05$, ** $p < 0.01$, *** $p < 0.001$, **** $p < 0.0001$.

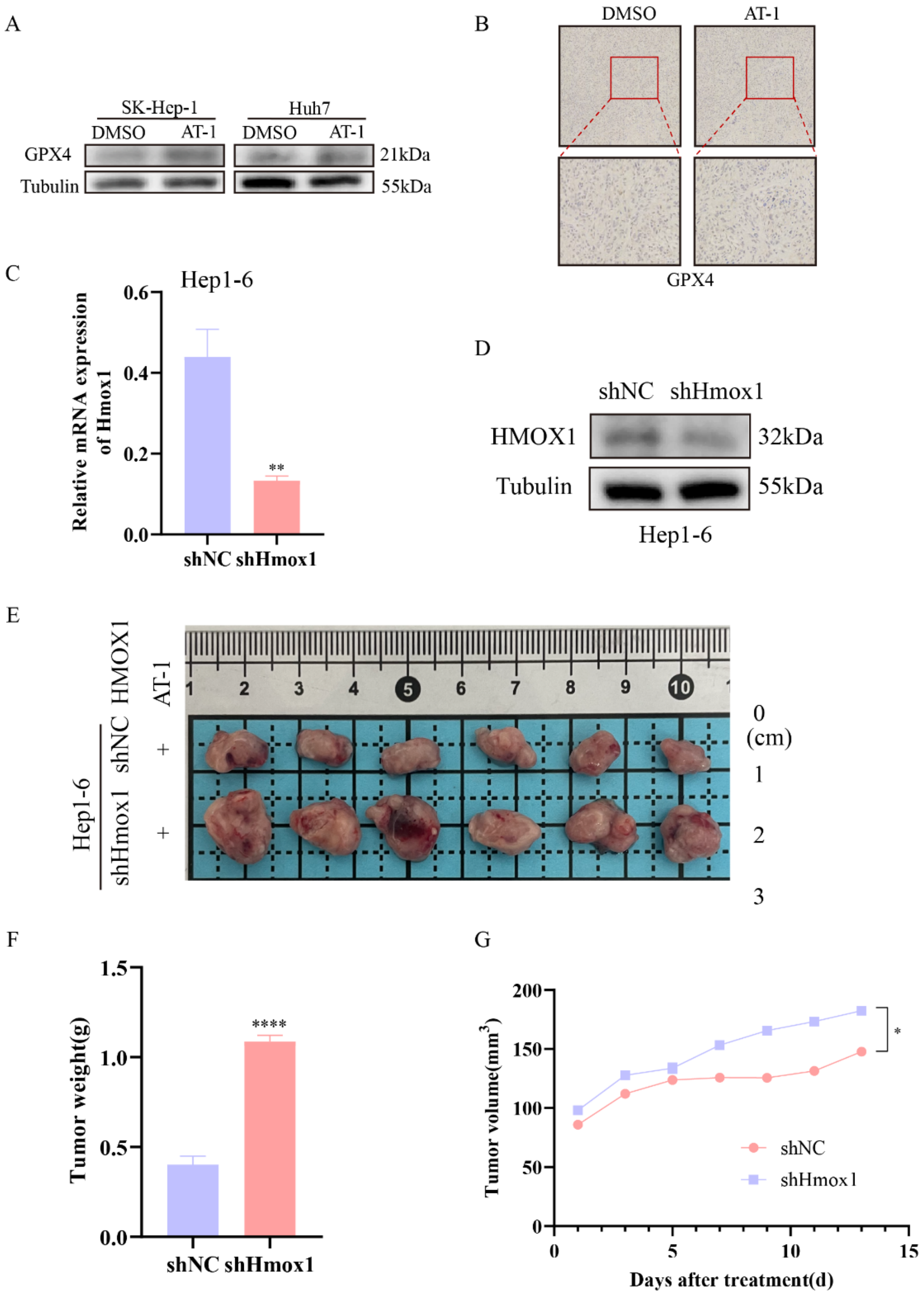

Supplementary Figure 2. HMOX1 knockdown attenuates the in-vivo anti-tumor activity of at-1 in a C57BL/6 syngeneic model, while GPX4 remains unchanged

(A) Immunoblot of GPX4 in SK-Hep-1 and Huh7 cells treated with DMSO or AT-1 (24 h); Tubulin as loading control. GPX4 levels are largely unchanged. (B) Representative IHC for GPX4 in xenograft tumors from DMSO- and AT-1–treated

groups (scale bar, 200 μm). (C) qPCR validation of Hmox1 knockdown in Hep1-6 cells; values normalized to shNC. (D) Immunoblot verification of Hmox1/HMOX1 protein levels in Hep1-6 cells (Tubulin control). (E) Representative excised tumors at endpoint from shNC and shHmox1 Hep1-6 cohorts. Beginning 7 days after inoculation, all mice received AT-1 (25 mg/kg, i.p., once daily) for 14 days. (F) Endpoint tumor weight. (G) Tumor-growth curves. Data are mean ± SEM; $n = 6$ mice per group for (E–G) and $n \geq 3$ independent experiments for (A–D). Statistics: unpaired two-tailed t-test for (C) and (F); two-way ANOVA with post-hoc correction for (G). Significance: * $p < 0.05$, ** $p < 0.01$, *** $p < 0.001$, **** $p < 0.0001$. Scale bar: 100 μm.
